# Autoresuscitation (Lazarus phenomenon) after termination of cardiopulmonary resuscitation - a scoping review

**DOI:** 10.1186/s13049-019-0685-4

**Published:** 2020-02-26

**Authors:** Les Gordon, Mathieu Pasquier, Hermann Brugger, Peter Paal

**Affiliations:** 1 Department of Anaesthesia, University Hospitals Morecambe Bay Trust, Royal Lancaster Infirmary, Lancaster, UK; 2 International Commission for Mountain Emergency Medicine (ICAR MEDCOM), Zermatt, Switzerland; 3 Emergency Department, Lausanne University Hospital, Lausanne, Switzerland; 4 Institute of Mountain Emergency Medicine, EURAC research, Bolzano, Italy; 5 Department of Anaesthesiology and Intensive Care, Hospitallers Brothers Hospital, Paracelsus Medical University, Salzburg, Austria

**Keywords:** Autoresuscitation, Cardiopulmonary resuscitation, Emergency medicine, Hyperventilation, Lazarus phenomenon, Resuscitation, Resuscitation orders

## Abstract

**Background:**

Autoresuscitation describes the return of spontaneous circulation after termination of resuscitation (TOR) following cardiac arrest (CA). We aimed to identify phenomena that may lead to autoresuscitation and to provide guidance to reduce the likelihood of it occurring.

**Materials and methods:**

We conducted a literature search (Google Scholar, MEDLINE, PubMed) and a scoping review according to PRISMA-ScR guidelines of autoresuscitation cases where patients undergoing CPR recovered circulation spontaneously after TOR with the following criteria: 1) CA from any cause; 2) CPR for any length of time; 3) A point was reached when it was felt that the patient had died; 4) Staff declared the patient dead and stood back. No further interventions took place; 5) Later, vital signs were observed. 6) Vital signs were sustained for more than a few seconds, such that staff had to resume active care.

**Results:**

Sixty-five patients with ROSC after TOR were identified in 53 articles (1982–2018), 18 (28%) made a full recovery.

**Conclusions:**

Almost a third made a full recovery after autoresuscitation. The following reasons for and recommendations to avoid autoresuscitation can be proposed: 1) In asystole with no reversible causes, resuscitation efforts should be continued for at least 20 min; 2) CPR should not be abandoned immediately after unsuccessful defibrillation, as transient asystole can occur after defibrillation; 3) Excessive ventilation during CPR may cause hyperinflation and should be avoided; 4) In refractory CA, resuscitation should not be terminated in the presence of any potentially-treatable cardiac rhythm; 5) After TOR, the casualty should be observed continuously and ECG monitored for at least 10 min.

## Introduction

Autoresuscitation describes the return of spontaneous circulation (ROSC) after termination of resuscitation (TOR) following cardiac arrest (CA), when resuscitation has been attempted but has been deemed unsuccessful and abandoned [1]. It was first described in 1982 [2] and has been seen in out-of-hospital and in-hospital situations. It is sometimes called the “Lazarus Phenomenon” or “Lazarus Syndrome” after Lazarus, who was raised from the dead after 4 days by Jesus. The actual incidence of autoresuscitation is unknown but it is not rare, as surveys have shown that 37–50% of intensive care or prehospital emergency physicians have encountered it in clinical practice [3–6]. This means that there may be many unreported cases, since there are ≈1900 Intensive Care consultants in the UK alone. It is believed that the condition is grossly under-reported, partly because of fear of legal repercussions [7–9]. The implications of even a few reports of autoresuscitation are significant, not only because it can cause dismay and distress to healthcare professionals, bystanders and family, but also because delayed ROSC could lead to questions being asked about whether resuscitation had been conducted properly and whether it was stopped prematurely [10–13]. Personnel delivering resuscitation should know about the existence of autoresuscitation before being confronted with it [4, 14]. It was therefore decided to review the reported cases of autoresuscitation in patients undergoing CPR, to identify any factors that may contribute to it, and highlight changes in practice that could potentially reduce the likelihood of it occurring. This article maps the evidence about autoresuscitation, identifies the main theories and knowledge gaps, and proposes guidance on issues during resuscitation, and when confirming death, that could have a bearing on whether autoresuscitation will occur.

## Methods

Cases of autoresuscitation were identified and selected through a scoping literature review. This method was chosen as it was felt to be most appropriate for identifying and mapping the available evidence on a very specific topic with a low-evidence base, when it is still unclear what other, more specific questions regarding its aetiology should be posed, and where evidence is still emerging [15]. We complied as much as possible with the Prisma-ScR scoping review checklist and guidelines [16]. We did not establish nor publish a priori a protocol for this study, and to our knowledge, there is not one currently in existence. For the purposes of this review, case reports conformed to all of the following criteria: 1) CA occurred from any cause; 2) Resuscitation attempts were performed (Basic Life Support or Advanced Life Support) for any length of time; 3) A point was reached when it was felt that either the patient had actually died (typically, persistent asystole) or a refractory arrhythmia occurred e.g. PEA, VF, that was not felt to be amenable to treatment; 4) Staff then declared the patient to be dead and stood back. No further interventions took place; 5) Later, signs of life were observed e.g. respiratory movements, electrocardiogram (ECG). Importantly, cases with transient ECG resumption compatible with cardiac output but no pulse were excluded. 6) The signs of life were sustained for more than a few seconds, such that staff had to resume active patient care. Patients who were expected to die, and therefore did not receive full Basic or Advanced Life Support, have not been included in this review. In addition, spontaneous reversion of VF to sinus rhythm with brief or no resuscitation attempts has been well documented for > 60 years [17–20] and is also not included.

We searched the literature up to 20th August 2019 with Google Scholar, MEDLINE and PubMed using the following terms alone and in combination: “Lazarus Phenomenon”; “Lazarus Syndrome”; “Autoresuscitation” or “Auto-resuscitation”. The composite search string used was (“lazarus phenomenon” OR “lazarus syndrome” OR “autoresuscitation” OR “auto-resuscitation”) for Google Scholar and ((“lazarus phenomenon”[All Fields] OR “lazarus syndrome”[All Fields]) OR “autoresuscitation”[All Fields]) OR “auto-resuscitation”[All Fields] for MEDLINE and PubMed. Articles of any type were included in this review, e.g. case reports and series; cohort and prospective studies and other systematic reviews, provided that they had been published in peer-reviewed journals. To expand the number of relevant retrieved articles, we then used two further search procedures. By placing the title of each article retrieved into the search field, we used the options to seek “Related articles” in Google Scholar and “Similar articles” in MEDLINE and PubMed. Articles in English, French, German, Russian, Spanish and Turkish were included. Finally, we searched the bibliography of the retrieved articles for additional articles that had not been picked up by the original searches.

The following data were collected for every case: age, sex, duration of resuscitation, rhythm when resuscitation was abandoned (e.g. asystole, pulseless electric activity (PEA), ventricular fibrillation (VF)), and the time when ROSC was first noticed. Outcome parameters included the cause of death or survival to hospital discharge (as appropriate) [21] and the neurological outcome (Cerebral Performance Category (CPC)) [22].

One author (LG) performed the literature search and built the database. In cases of doubt regarding the inclusion of a given case, the case was independently assessed for eligibility by the co-authors. All reports that clearly described a case of autoresuscitation were included, no matter how much detail was included. Descriptive statistics included frequencies, means and standard deviations, or median and interquartile range (IQR) to help better understand the scope of the problem. Groups were compared using Pearson’s Chi-square or Fischer’s exact tests, Student’s t-test, or the Wilcoxon rank-sum test as appropriate. The data retrieved from the database were exported to Stata version 14 (Stata Corporation, College Station, TX). A bilateral *p*-value < 0.05 was considered to indicate a significant difference.

## Results

**Fig. 1:**
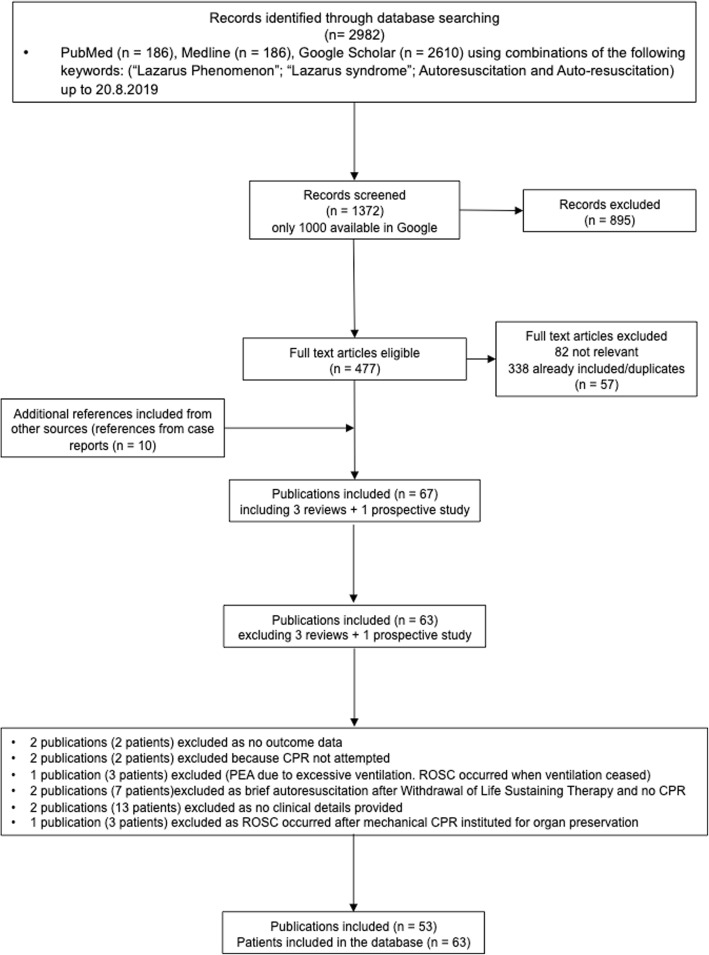
Flowchart of patient selection

The literature search generated 1372 publications, 53 references with 63 patients for which outcome was available were included (Fig. 1). All the references were retrospective case reports of which five [2, 13, 23–25] described two patients, and one [26] described a series of five patients (Additional file 1: Table S1). Brief clinical details of the cases included in this review are reported in Additional file 2. We also included three published reviews and a prospective study (Additional file 3) in the qualitative synthesis.

We included 34 out-of-hospital and 29 in-hospital CAs. The mean age of the patients was of 61 ± 24 (range 9 months – 97 years), the majority (68%) occurred in patients > 60 years old. The rhythm when CPR was first abandoned was asystole in 38 (70%) of the 54 patients for which this information was available (12 survivors; 26 non-survivors), PEA in 12/54 (22%) (3 survivors; 9 non-survivors), and VF in 4/54 (7%) (survivors only). Median duration of resuscitation was 30 min (IQR 18–40; range 0–90 min). Resuscitation attempts were performed for < 20 min (the time often recommended before abandoning resuscitation) [27–32] in 14 of 63 cases (22%). Signs of life were first noticed within 5 min in 30/63 cases (47%) cases and at 6–10 min in 14/63 cases (22%) (median of 5 min (IQR 3–10; range 0–220 min)) after resuscitation had been stopped. In the remaining cases, signs of life were not noticed until later or not recorded. In some cases, signs of life were only observed several hours after “death” was supposed to have occurred. Out of the 63 patients, 22 (35%) survived to hospital discharge, most (18; 82%) with good neurological outcome (Table 1). Forty-one patients died, the majority whilst still in hospital (Table 2) due to severe hypoxic brain damage or cardiac problems in most cases (28/41; 68%), while 4/41 (10%) initially fully recovered from arrest (CPC 1 or 2) but died due to other causes. Data on outcome was missing for two additional patients (Table 3). There was no difference between survivors and non-survivors regarding age (*p* = 0.18), duration of resuscitation (*p* = 0.47), and signs of life first noticed (*p* = 0.80). The rhythm when resuscitation was abandoned was associated with survival, as all four patients with VF survived, compared with 12/38 (32%) and 3/12 (35%) of those with asystole and PEA, respectively (*p* = 0.017).

**Table 1:** Patients who survived autoresuscitation to hospital discharge (*n* = 22)

Case	Reference	Age (y)	In-hospital (I) 14 Prehospital (P) 8	Rhythm when resuscitation abandoned	Duration of resuscitation (min)	Signs of life first noticed (min)	Delay before death (s,h,d,m)	CPC
		56 ± 24 (range 0.9–84)		Asystole 12 (55%); VF 4 (18%); PEA 3 (14%); Not stated 3 (14%)	30 (IQR 25–47.5; range 5–75)	5.5 (IQR 3.5–10; range 1–20)		CPC 1–2
1)	[2]	68	I	VF	75	20	–	1
2)	[34]	67	I	“Abnormal”	30	4	–	1
3)	[35]	81	I	Asystole	5	2	^f^	1
4)	[36]	50	I	Asystole	30	2	8 m	1
5)	[59]	36	I	PEA	25	3	–	1
6)	[60]	84	P	VF	15	5	–	1
7)	[61]	27	P	Asystole	> 25^a^	1	–	1
8)	[62]	21	I	Asystole	30	10	–	1
9)	[63]	80	I	Asystole	20	5	–	1
10)	[64]	66	I	Asystole	18	10	–	1
11)	[65]	66	P	PEA	45	5	–	1
12)	[66]	83	I	NS	60	7	–	1
13)	[67]	70	I	Asystole	34	8	–	1
14)	[23]	66	P	VF	> 30^a^	NS^b^	–	2 (right sided hemiparesis)
15)	[23]	71	I	Asystole	35	NS^b^	–	1
16)	[68]	63	P	Asystole	70	NS ^d^	–	1
17)	[69]	51	I	Asystole	61	3.5	–	2 (R arm weakness and partial amnesia)
18)	[70]	54	P	PEA	50	NS^d^	–	1
19)	[71]	69	I	Asystole	25	10	^e^	3/Hypoxic brain damage
20)	[8]	47	P	VF	26	15	3 m	3/Hypoxic brain damage
21)	[72]	0.92	I	Asystole	NS^c^	15	6 weeks	3/Died progressive cardiomyopathy
22)	[73]	1.5	P	NS	NS^a^	6	–	3/Severe disability at 1y

*ALS* denotes advanced life support, *BLS* basic life support, *CPC* cerebral performance category, *ED* emergency department, *IQR* interquartile range, *PEA* pulseless electrical activity, *NS* not stated, *VF* ventricular fibrillation; ^a^BLS on scene + ALS in ambulance + ED; duration was considered =25 min and = 30 min for the analysis; ^b^ “moments”; ^c^ Intermittent over hours; ^d^ < 10 min; ^e^ = Alive at end of case report; ^f^ “few days

**Table 2:** Patients who did not survive autoresuscitation (*n* = 41)

Case	Reference	Age	In-hospital (I) 15Prehospital (P) 26	Rhythm when resuscitation abandoned	Duration of resuscitation (min)	Signs of life first noticed (min)	Delay before death (s,h,d,m)	CPC (if available)/Outcome or Cause of death
		64 ± 24 (range 0.75–97)		Asystole 26 (63%); PEA 9 (22%); VF 0 (0%); Not stated 6 (15%)	30 (IQR 17–40; range 0–90)	5 (IQR 2.5–10; range 0–220)		
1)	[2]	84	P	Asystole	17	^d^	6d	3/Hypoxic brain damage
2)	[2]	67	P	Asystole	20	^c^	15d	3/Hypoxic brain damage
3)	[7]	80	I	Asystole	30	5	2d	3/Hypoxic brain damage
4)	[10]	65	P	Asystole	35	20	5d	4/Hypoxic brain damage
5)	[11]	81	P	Asystole	13	NS^c^	20 h	3/Cardiogenic shock
6)	[13]	65	P	NS	35	20	4d	4/Myocardial infarction
7)	[13]	83	P	Asystole	23	33	6.5 h	4/Myocardial infarction
8)	[26]	97	P	Asystole	16	3	2 m	Died without regaining consciousness
9)	[26]	30	P	PEA	31	6	88 m	Died without regaining consciousness
10)	[26]	63	P	PEA	12	3	26 h	Died without regaining consciousness
11)	[26]	91	P	PEA	16	3	15 m	Died without regaining consciousness
12)	[26]	61	P	PEA	18	8	3 m	Died without regaining consciousness
13)	[37]	53	I	Asystole	46	2	34d	4/Died (multi-organ failure)
14)	[74]	89	I	Asystole	18	5	7 h	Died. Another cardiac event.
15)	[24]	0.75	P	Asystole	NS^d^	0.5	Several days.	Died (WLST).
16)	[24]	3	I	Asystole	25	1	40 m	Died (WLST)
17)	[75]	64	I	NS	20	15	1 h	Died. Refractory hypotension.
18)	[76]	76	I	Asystole	30	5	24 h	3/Died. Hypoxic brain damage.
19)	[77]	44	P	PEA	80	5	NS^e^	3/Palliative care only
20)	[78]	87	I	Wide complexes^g^	15	Immediately	12d	4/Died
21)	[79]	63	I	Asystole	12	3	12d	Died. Sepsis.
22)	[80]	59	I	NS^b^	15	2	30 m	Died.
23)	[81]	67	P	PEA	55	2	22 h	1/Died from severe comorbidity.
24)	[82]	10	P	PEA	> 40^f^	2	Short time after	5 (WLST)
25)	[83]	94	I	PEA	40	2.5	18d	1/Died multi-organ failure
26)	[84]	65	P	Asystole	55	40	13d	Died without regaining consciousness
27)	[85]	35	P	NS	88	NS^c^	50 m	Died. Another cardiac event.
28)	[86]	83	P	Asystole	90	10	12d	Died. Pneumonia.
29)	[87]	85	P	Asystole	34	2	25 m	Died. Another cardiac event.
30)	[88]	62	I	Idioventricular	40	5	34 m	Died without regaining consciousness
31)	[89]	55	P	Asystole	30	7	3d	5/Died. Hypoxic brain damage.
32)	[25]	63	P	Asystole	40	10	10 m	Died without regaining consciousness
33)	[25]	78	P	Asystole	41	2	20 m	Died without regaining consciousness
34)	[90]	78	P	Asystole	31	220	19 h	Died without regaining consciousness
35)	[91]	85	P	Asystole	0	6	48 h	4/Died (WLST)
36)	[92]	69	P	Asystole	40	180	10d	Died without regaining consciousness
37)	[93]	46	P	Asystole	30	60	NS (hours)	2/Died from cardiogenic shock
38)	[94]	83	I	Asystole	15	10	2 h	Died without regaining consciousness
39)	[95]	25	I	NS	40	5–7	4 h	Died without regaining consciousness
40)	[96]	67	I	Asystole	49	5	9d	1/Died of massive pulmonary embolism
41)	[97]	75	I	Asystole	23	5	Several days	Died without regaining consciousness

*CPC* cerebral performance category, *d* days, *ED* emergency department, *h* hours, *IQR* interquartile range, *PEA* pulseless electrical activity, *m* months, *NS* not stated, *s* seconds, *VF* ventricular fibrillation; *WLST* Withdrawal of Life Sustaining Therapy; ^a^ age in 40’s; ^b^ possible/probable PEA, interpreted as PEA in the analysis; ^c^ Exact time when signs of life were first noted is not stated but the authors stated that it was “several” or “a few minutes”; ^d^ Return of signs of life not stated but > 10 min (considered as =10 min for the analysis); ^e^ = Alive at end of case report; ^f^ considered = 40 for the analysis; ^g^ no more information, considered as a missing data for the analysis

**Table 3:** Unknown final outcome of autoresuscitation (*n* = 2)

Case	Ref	Age	Rhythm when resuscitation abandoned	Duration of resuscitation (min)	Signs of life first noticed (min)	Delay before death	CPC (if available)/Outcome or cause of death
1)	[98]	93	NS	6	5	NS	Outcome not stated
2)	[99]	40	Asystole	45	30	NS	Outcome not stated

*CPC* denotes cerebral performance category, *NS* not stated

The definitions of autoresuscitation in published case reports vary but all contain the same two elements: 1) attempted and abandoned resuscitation attempts following CA, 2) subsequent ROSC without medical intervention (Additional file 4).

## Discussion

The most important finding is that about 30% of patients made a good recovery after death had been diagnosed, so autoresuscitation is of major significance and puts a focus on resuscitation practice, the decision to terminate resuscitation and diagnosis of death. Although the biblical Lazarus rose from the dead without resuscitation attempts, there are no case reports of it occurring in someone who has died without CPR performed beforehand [9, 33]. Almost all cases of autoresuscitation occurred after CPR following non-traumatic CA; there is only one report following traumatic CA [34] and four others associated with major haemorrhage [26, 35–37]. Therefore, it has been suggested that autoresuscitation is due to the medical interventions that were performed during resuscitation, but their effectiveness was delayed for some reason [38]. Cases occurring in patients who have undergone withdrawal of life-sustaining treatment were short-lived [39], apart from the subgroup of intended organ donors in which death was thought to have occurred and the circulation was subsequently maintained by artificial means [40, 41]. It is essential to consider possible mechanisms of autoresuscitation because this has potential implications for the way resuscitation is performed. A clear mechanism for autoresuscitation has been identified in only a few cases. The pathophysiologic factors (possibly in combination) that are thought to contribute to autoresuscitation are listed in Table 4 and derived from the conclusions of the authors of the published case reports and studies. Autoresuscitation has been reported more frequently in adults than children [3, 33].

**Table 4:** Proposed autoresuscitation mechanisms and recommendations based on case reports to reduce the likelihood of it occurring

factor	Proposed Mechanism	Actions that might reduce the Likelihood of Autoresuscitation occurring
Poor controlled ventilation techniques		
1) Air trapping in the lungs causing hyperinflation	Caused by high tidal volume or rapid ventilation rates with insufficient time for exhalation. Releasing the positive intra-thoracic pressure will enable venous return to resume and restore the circulation [24, 38, 59, 64, 65, 74–79, 86, 98, 100–102]. Effect more pronounced in hypovolaemia [37, 64] and pre-existing obstructive airways disease, especially if not managed correctly [9, 59, 103].	Avoid excessive ventilation (rate, tidal volume, or both) Exclude hyperinflation as a reversible cause of Pulseless Electrical Activity (PEA) by stopping ventilation and disconnecting the bag
2) High intrathoracic pressure	Delays injected CPR drugs from reaching the heart and allows drugs to accumulate peripherally. Stopping positive airway pressure allows drugs to reach the heart resulting in beneficial effects [65, 80, 86].	
3) Hyperventilation	Deleterious effects on coronary perfusion pressure (CPP) [104].	
Delayed drug effects	In profound acidosis or impaired drugs delivery via peripheral or intraosseous lines [77–79].	
CPP as low as 15 mmHg can produce Return of Spontaneous Circulation after asystole	Intrinsic vasomotor function of capacitance and resistance blood vessels may maintain CPP so that even when resuscitation has ceased, CPP may be high enough to restart the heart [105].	Careful consideration before terminating resuscitation if vasopressor infusions and/or mechanical ventilation are used
Return of myocardial function following Termination of Resuscitation (TOR)	Myocardial reperfusion due to spontaneous dislodging of endovascular plaque from a coronary artery [7, 10, 38]. Might also possibly allow spontaneous defibrillation in refractory VF [8, 23, 60].	
Premature TOR	Failure to appreciate that transient asystole can occur immediately after defibrillation [23].	Resuscitation should never be abandoned immediately after defibrillation.
	Resuscitation terminated prematurely before therapeutic measures could have adequate effect.	Careful consideration before terminating resuscitation especially if vasopressor infusions and/or mechanical ventilation are used.
	Untreated reversible causes e.g. acid-base balance; electrolyte imbalance; hypothermia [68].	Check for and correct all reversible causes of CA before considering TOR.
	TOR in the presence of a potentially treatable cardiac rhythm (refractory VF, PEA, broad complexes, bradycardia) and not asystole.	Caution about which cardiac rhythms are acceptable for terminating resuscitation as in 30% of autoresuscitation cases, TOR had occurred in the presence of some cardiac electrical activity (i.e. not asystole)
	TOR too soon after resuscitation started	Careful consideration of how long CPR has been employed before TOR
Procedural	Unobserved minimal vital signs (e.g. pseudo-PEA) due to clinician oversight [38, 81]. Misdiagnosis of death, perhaps due to failure to fully examine patient prior to declaring death.	A 10 min observation period with ECG is generally more appropriate than 5 min following TOR [2, 7–11, 14, 26, 60–64, 66, 76, 79, 81, 94, 95, 106, 107]. After the decision has been made to terminate resuscitation, chest compressions should not be restarted The possibility of autoresuscitation should not affect the decision about when to terminate resuscitation
Resuscitation may exacerbate acute internal bleeding leading to hypovolaemic arrest	When resuscitation is stopped, the cardiovascular system stabilises [36].	Observe the patient after TOR for 10 min.

Death may be defined as the irreversible cessation of vital functions, including absence of circulation, spontaneous breathing, and whole-brain death when no confounding factors are present [27, 42, 43]. Doctors therefore diagnose death based on the absence of functions that are fundamental for life. The Academy of UK Medical Royal Colleges guidelines specify that there should be an absence of heart sounds, a central pulse on palpation, pupillary responses to light, corneal reflexes, and any motor response to supra-orbital pressure before confirming death [42]. In an advanced care setting, these findings can be supplemented with: asystole on a continuous ECG, absence of pulsatile flow with intra-arterial monitoring or the absence of contractile activity using echocardiography [42]. Although patients exhibiting the above clinical findings are assumed to have passed the “point of no return” and become unsalvageable, in fact death is not an instantaneous event but takes place over time. Sporadic ECG activity in the absence of a circulation can occur for many minutes after death is diagnosed [1, 33, 39, 44], and this can confound the Academy’s use of asystole as an indicator of death. Thus, an essential requirement when defining autoresuscitation is the presence of a circulation, because death determination depends on the cessation of circulation, not just of cardiac electrical activity [45]. In addition, a recent animal study indicated that sporadic cortical neuronal activity may be present for 2 hours following cardiac arrest [46]. If ECG activity resumes, it is important to establish if it is in isolation or whether ROSC has occurred [45]. Importantly, arterial pulselessness and asystole for a short period, e.g. immediately after defibrillation [23, 47], cannot reliably establish that irreversible cessation of cardiac and neurological function has occurred [48]. Finally, several autoresuscitation case-reports have occurred in the presence of a discernible cardiac rhythm (refractory VF or PEA, wide QRS complexes, extreme bradycardia) i.e. not asystole. Therefore, caution is advised before abandoning resuscitation in the presence of an ECG that is potentially treatable or compatible with life [26].

The Academy also advises that the person responsible for confirming death should observe the patient for “a minimum of five minutes to establish that irreversible cardiorespiratory arrest has occurred” [42]. The observation period after TOR is crucial and could leave carers open to the criticism that resuscitation was terminated prematurely [10] if an adequate period of observation after TOR is not employed. Importantly, the 5 min observation period [42] will potentially miss almost half of the autoresuscitation cases identified in this review. Although a care provider will potentially always be open to the charge that the resuscitation efforts were ended prematurely, regardless of the period of observation following termination of efforts, it is unreasonable to recommend that a patient is observed for a prolonged period of time after TOR solely in case autoresuscitation occurs. There has to be a balance between stopping the observation period prematurely at one extreme and waiting for a protracted period of time at the other. Our analysis of the case reports suggests that increasing from 5 to 10 min will increase the number of cases of autoresuscitation that will be picked up from 47% in ≤5 min to 69% within 10 min, and this increase is advocated by many of the authors of the case reports.

### Recommendations

It has been calculated that a study with 95% confidence interval and 80% statistical power would need to document zero cases of autoresuscitation among 10,516 patients just to “rule out” even a rather high autoresuscitation rate of one in 1000 deaths [49]. Therefore, it is unlikely that a formal study that includes sufficient numbers of patients will ever be conducted. Consequently, it is necessary to review the factors that have been identified in actual cases and propose measures that can be taken to reduce the likelihood of autoresuscitation occurring. A summary of these, the rationale underpinning them and the relevant references are detailed in Table 4. These have been derived from the conclusions of the authors of each individual case report. There are five principal points to consider:

1. Resuscitation attempts should be continued for at least 20 min. Although this is now standard practice, in 22% of autoresuscitation cases, resuscitation was performed for less than this.
2. Attempt to avoid high intrathoracic pressure and hyperinflation during ventilation, as these are believed to be the underlying mechanism in many of the autoresuscitation case reports. In practice, this means gentle manual ventilation at no more than 12 breaths/minute. In patients with a history of chronic lung disease, it may be helpful to periodically disconnect the breathing circuit to ensure that the lungs are fully deflated before recommencing ventilation to avoid hyperinflation of the chest and thus diminished venous return to the heart.
3. As transient asystole can occur after defibrillation, careful consideration should be given before abandoning resuscitation immediately after an unsuccessful defibrillation attempt.
4. In refractory cardiac arrest, it might be unwise to terminate resuscitation in the presence of any potentially treatable cardiac rhythm, as this can become a perfusing rhythm. If an ECG monitor is not available and the cardiac rhythm is unknown (e.g. as in some Search and Rescue teams staffed by lay people), attempts should be made to get healthcare professional advice before terminating resuscitation.
5. After termination of resuscitation, consider extending the patient observation period from five to 10 min with ECG monitoring. No rules are going to exclude all cases of autoresuscitation but extending the observation period from five to 10 min will increase the safety margin from 47 to 69%. It is important to remember that the quoted times to recognition that the patient was still alive recorded in the case reports are generally not the times that autoresuscitation actually occurred. As explained above, this is because in most cases, patient monitoring was stopped when death was declared, and it was only when something changed e.g. the patient was noticed to be breathing, that it was clear that they were not dead.

### Limitations

This is a scoping review and not a systematic review. More databases could have been accessed. Also, there may be more reports because some older papers published in print are now unavailable. The quality of reporting was generally low (case reports or letters to the editor), and often contained too few data to be included in this review [50]. In most reports, continuous monitoring was switched off once resuscitation had been abandoned, so the time when signs of life were detected is not an accurate guide to when ROSC occurred. This explains at least some of the cases where the time interval from TOR to signs of life detected was prolonged. Given the scarcity of data with autoresuscitation it may be necessary to make recommendations, which are mainly based on case reports and series. Nevertheless, we are not aware of any better method than this scoping review to assess autoresuscitation. Autoresuscitation may be a concern in potential organ donors. This specialist area is covered elsewhere, so it was felt inappropriate to draw it into this discussion [3, 45, 51–58].

We have attempted to overcome some of the limitations imposed by language restrictions. Another potential limitation is that the search strategy focussed on papers in which the title indicated that the theme of the paper was about autoresuscitation, Lazarus, etc. This was to avoid identifying the many papers in which autoresuscitation is mentioned but only as part of a wider discussion about resuscitation. The recommendations have been based on a small sample size.

## Conclusions

Almost a third of the patients made a full recovery after autoresuscitation. This emphasises resuscitation should be terminated with caution. The following reasons for and recommendations to avoid autoresuscitation can be given: 1) In asystole with no reversible causes, resuscitation efforts should be continued for at least 20 min; 2) Resuscitation should not be abandoned immediately after unsuccessful defibrillation, as transient asystole can occur after defibrillation; 3) Excessive ventilation during resuscitation may cause hyperinflation and should be avoided; 4) In refractory cardiac arrest, resuscitation should not be terminated in the presence of any potentially-treatable cardiac rhythm; 5) After TOR, the casualty should be observed continuously and ECG monitored for at least 10 min.

## Supplementary information

**Additional file 1. Table S1.** Year of publication, number of autoresuscitation cases and references reported in this review

**Additional file 2.** Published autoresuscitation case reports with brief clinical details

**Additional file 3.** Published reviews and prospective studies

**Additional file 4.** Published definitions of autoresuscitation
