# Supplementary material for: Autoresuscitation (Lazarus phenomenon) after termination of cardiopulmonary resuscitation - a scoping review
Source: Scand J Trauma Resusc Emerg Med. 2020 Feb 26;28:14. doi: 10.1186/s13049-019-0685-4 (PMC7045737; doi:10.1186/s13049-019-0685-4)
Supplement: Supplementary file 1 — Additional file 1. Table S1. Year of publication, number of autoresuscitation cases and references reported in this review [file 13049_2019_685_MOESM1_ESM.docx]

**Supplemental Table 1.** Year of publication, number of autoresuscitation cases and references reported in this review

| **Year published** | **No cases** | **Reference** |
| --- | --- | --- |
| 1982 | 3 | ([2](#_ENREF_2)) |
| 1982 | 1 | ([63](#_ENREF_63)) |
| 1991 | 1 | ([59](#_ENREF_59)) |
| 1991 | 1 | ([75](#_ENREF_75)) |
| 1993 | 1 | ([78](#_ENREF_78)) |
| 1993 | 1 | ([96](#_ENREF_96)) |
| 1994 | 1 | ([67](#_ENREF_67)) |
| 1996 | 2 | ([22](#_ENREF_22)) |
| 1996 | 1 | ([88](#_ENREF_88)) |
| 1997 | 1 | ([70](#_ENREF_70)) |
| 1997 | 1 | ([84](#_ENREF_84)) |
| 1998 | 1 | ([7](#_ENREF_7)) |
| 1998 | 1 | ([95](#_ENREF_95)) |
| 1999 | 1 | ([76](#_ENREF_76)) |
| 1999 | 1 | ([80](#_ENREF_80)) |
| 2001 | 1 | ([61](#_ENREF_61)) |
| 2001 | 1 | ([64](#_ENREF_64)) |
| 2002 | 1 | ([10](#_ENREF_10)) |
| 2003 | 1 | ([35](#_ENREF_35)) |
| 2004 | 1 | ([11](#_ENREF_11)) |
| 2004 | 1 | ([82](#_ENREF_82)) |
| 2005 | 2 | ([13](#_ENREF_13)) |
| 2005 | 1 | ([79](#_ENREF_79)) |
| 2006 | 1 | ([66](#_ENREF_66)) |
| 2006 | 1 | ([89](#_ENREF_89)) |
| 2007 | 1 | ([8](#_ENREF_8)) |
| 2007 | 1 | ([90](#_ENREF_90)) |
| 2010 | 1 | ([60](#_ENREF_60)) |
| 2010 | 1 | ([86](#_ENREF_86)) |
| 2010 | 1 | ([93](#_ENREF_93)) |
| 2011 | 1 | ([69](#_ENREF_69)) |
| 2011 | 2 | ([23](#_ENREF_23)) |
| 2011 | 1 | ([81](#_ENREF_81)) |
| 2011 | 1 | ([85](#_ENREF_85)) |
| 2012 | 1 | ([37](#_ENREF_37)) |
| 2012 | 1 | ([83](#_ENREF_83)) |
| 2012 | 1 | ([94](#_ENREF_94)) |
| 2013 | 1 | ([74](#_ENREF_74)) |
| 2013 | 1 | ([87](#_ENREF_87)) |
| 2013 | 2 | ([24](#_ENREF_24)) |
| 2014 | 1 | ([62](#_ENREF_62)) |
| 2015 | 1 | ([36](#_ENREF_36)) |
| 2015 | 1 | ([58](#_ENREF_58)) |
| 2015 | 1 | ([72](#_ENREF_72)) |
| 2015 | 1 | ([92](#_ENREF_92)) |
| 2016 | 1 | ([71](#_ENREF_71)) |
| 2016 | 1 | ([77](#_ENREF_77)) |
| 2017 | 1 | ([34](#_ENREF_34)) |
| 2017 | 5 | ([25](#_ENREF_25)) |
| 2017 | 1 | ([65](#_ENREF_65)) |
| 2017 | 1 | ([91](#_ENREF_91)) |
| 2018 | 1 | ([68](#_ENREF_68)) |
| 2018 | 1 | ([73](#_ENREF_73)) |
