# Supplementary material for: Autoresuscitation (Lazarus phenomenon) after termination of cardiopulmonary resuscitation - a scoping review
Source: Scand J Trauma Resusc Emerg Med. 2020 Feb 26;28:14. doi: 10.1186/s13049-019-0685-4 (PMC7045737; doi:10.1186/s13049-019-0685-4)
Supplement: Supplementary file 2 — Additional file 2. Published autoresuscitation case reports with brief clinical details [file 13049_2019_685_MOESM2_ESM.docx]

## **Supplemental file 2. Published autoresuscitation case reports with brief clinical details**

The majority of the cases listed were reported as individual reports and most have been included in the three published retrospective reviews.^1-3^ Enough details were reported to include two further cases that occurred after withdrawal of life-sustaining therapy.^4^ For completeness, four case reports of autoresuscitation have been published in which the patient had a recognised reversible cause of cardiac arrest so that CPR may have been terminated prematurely in these cases.^5-8^ In a sense, these are not autoresuscitation since ROSC is the natural conclusion of the correct management. Current best practice would dictate that these should be treated before abandoning resuscitation. In addition, a case report describes possible fat embolism from fractured neck of femur.^9^ The evidence is very weak for fat embolism as the cause in this report, particularly as the patient was old and had significant chronic cardiorespiratory disease and it is more likely to have been due to air-trapping during resuscitation, as pointed out by the authors. In the summaries, Advanced Life Support (ALS) interventions refers to full resuscitative measures (i.e. chest compressions, defibrillation, drugs as required especially epinephrine, etc.).

Case reports of autoresuscitation following failed termination of CPR in acute cardiac arrest (53 articles describing 63 patients)

BLS = Basic Life Support. ALS = Advanced Life Support

- 69y. F. Medical history of C2 odontoid fracture with quadriplegia, recurrent urinary tract infections, and end-stage renal disease on haemodialysis presented with lethargy and a fever of 39.3°C. Whilst undergoing haemodialysis, she became bradycardic and subsequently, pulseless (PEA) arrest. Resuscitation commenced, and she went into VF and received multiple defibrillations and ALS drugs. After 25 minutes without a result, the family who were present asked the medical team to cease resuscitation efforts. There were no palpable pulses and cardiac ultrasound confirmed no heart contractions. In addition, the patient had no corneal reflexes and no spontaneous breathing. She was pronounced dead and the family gathered round to grieve. Ten minutes later, the doctors were called back as the patient had started to breathe and had a palpable carotid pulse. The patient survived to discharge but had sustained hypoxic brain damage.^10^
- (3 cases reported). (1) 68y. F. Previous myocardial infarction (MI); cardiac failure; diabetes; hypertension; ventricular arrhythmias. Multiple medications. Ventricular fibrillation (VF) arrest. CPR. Return of spontaneous circulation (ROSC). Recurrent VF despite repeated defibrillation attempts and ALS drugs. Resuscitation abandoned after 75 minutes when it was felt that the VF was refractory. Patient subsequently became asystolic. Twenty minutes later, the patient was noted to be breathing and moving. ECG showed normal sinus rhythm and blood pressure was normal. Two further episodes of VF responded to defibrillation. The next day, the patient opened her eyes and was neurologically normal. Blood tests confirmed a myocardial infarction. Discharged from hospital. Died three months later in her sleep. (2). 84-year-old man. Coronary artery and peripheral vascular disease; diabetes; emphysema. Fell from his bicycle. Bystander cardiopulmonary resuscitation (CPR) started within two minutes. Emergency Medical Services (EMS) arrived five minutes later. No vital signs. VF. Full ALS care. CPR discontinued after 10 minutes when monitor showed asystole. Patient noted to be breathing in the mortuary. ECG showed normal sinus rhythm. Normal blood pressure. Patient had signs of brain damage and died 6 days later. (3). 67-year-old male. Alcoholic. Chest pain. Collapse. Bystander CPR. When EMS arrived, VF. ROSC achieved but after 20min, asystole developed, and CPR was discontinued. After ‘some minutes’, the patient started to breathe spontaneously and had a weak pulse. Hypotensive. ECG confirmed myocardial infarction. Patient survived for 15 days but had sustained hypoxic brain damage.^11^
- 80y. M. Intensive Care Unit (ICU) patient with pancreatitis and sepsis. Bradycardia → asystolic arrest. CPR. ALS. Epinephrine briefly effective in ROSC. 5-10 minutes later, asystole resumed. After the third episode, it was decided to abandon resuscitation because 30 minutes of CPR had been given + patient age and co-morbidity. ECG showed asystole. No recordable blood pressure. Ventilator left running. Five minutes later, ROSC had occurred. It started as bradycardia and the blood pressure rose. However, the patient died two days later.^12^
- 47y. M. Found unconscious in a public toilet. EMS arrived 3 minutes later. Time of arrest was unknown, but the patient was in PEA. CPR commenced. Full ALS care. Rhythm changed to VF (received 13 defibrillations). The VF changed to short runs of asystole. Transient ROSC occurred at 13 minutes. After 26 minutes with no response, resuscitation was abandoned. The last noted rhythm was VF. All monitors were disconnected but the tracheal tube was left in situ and the patient was placed into a body bag. Fifteen minutes after the end of resuscitation, a police officer opened the body bag to record observations of the body and noticed the patient was breathing spontaneously. Monitoring was recommenced. Normal sinus rhythm. Good blood pressure. Taken to hospital. ICU. Discharged from hospital but with severe neurological damage. Died three months later.^13^
- 65y. M. Found unconscious at home. BLS for six minutes until EMS arrived. Full ALS. CPR abandoned 35 minutes after the original arrest with continuous asystole. 20 minutes later, a police officer who had been called for a post-mortem investigation found the patient slightly moving. After the recovery of cardiac rhythm, ECG showed a sinus tachycardia and the signs of a myocardial infarction. The patient never recovered consciousness and died five days later. Medical negligence due to premature termination of CPR was alleged and forensic autopsy was performed about 14 h after death.^14^
- 81y. F. Collapsed in the street. EMS immediately on scene. Cardiac arrest (VF). Full ALS. Asystole occurred after the second defibrillation attempt. Resuscitation was terminated after 13 minutes. “A few minutes later”, the patient was heard making noises. On examination, she had a pulse of 60 and a blood pressure of 80. The patient was taken to hospital but died 20 hours later of cardiogenic shock. Importantly, the first doctor who had intervened to assist the patient in the reported case was accused of culpable homicide for having brought forward the patient’s death due to his untimely cessation of resuscitation techniques.^15^
- (2 cases reported). (1). 65y. M. Out-of-hospital cardiac arrest. 35 minutes after care started, death was confirmed because of persistent cardiopulmonary arrest as shown on the ECG monitor. Twenty minutes later, a police officer in charge of the investigation found in the clinic mortuary that the patient was moving. Breathing and cardiac activity had resumed. The patient died four days later. (2). 83y. F. Collapsed at a bus stop. Bystander CPR. EMS arrived six minutes later. Transient cardiac electrical activity occurred following defibrillation lasting a few seconds. The ECG then showed asystole again. After 17 minutes, the decision was taken to terminate resuscitation. The patient was transferred by ambulance to a body holding area. On arrival there, 33 minutes had passed since the death. In the elevator to the mortuary, the two policemen notice breathing movements. After uncovering the neck, they discovered weak pulsations and a breath was heard from the endotracheal tube.^16^
- 67y. M. High-energy thoracic trauma. Conscious at the scene. Immobilised. Given ketamine, midazolam, phenylephrine and tranexamic acid. Ventilated by face mask for transportation to hospital. In the ED, RSI performed. Successful intubation. L thoracostomy for flail chest. BP dropped to 54 systolic with tachycardia. Catecholamine and fluids infused. PEA. CPR. Reversible causes excluded. Second chest tube inserted on the R to exclude missed injuries. Ultrasound excluded intrathoracic and abdominal bleeding. Venous blood gas showed pH 6.96. Hb 105 g/L. Mild hypothermia. Mechanical CPR used but after 30 minutes, CO_2_ was undetectable, the ECG was becoming increasingly abnormal and the pupils were dilated. Resuscitation was abandoned. Four minutes later, ROSC occurred. Whole body CT showed multiple fractures including ribs and scapula and a contained rupture of the ascending aorta. Hb 54. Massive transfusion. Ventricular tachycardia spontaneously reverted to sinus rhythm. Patient taken to ICU where he remained for 18 days. Discharged from hospital after 8 weeks. Five months after the accident, recovery was almost full with only seldom short memory problems.^17^
- (Five cases reported). (1) 97y. F. Witnessed collapse in a nursing home. Initial rhythm was asystole and remained so. BLS performed. A pharyngeal tube was used for airway management. Neither defibrillation nor epinephrine were used. Resuscitation was abandoned after 16 minutes due to continuous asystole. Three minutes later, a junctional rhythm resumed accompanied by agonal breathing and a palpable carotid pulse. This lasted for two minutes after which, the patient died. (2) 30y. F. The initial rhythm was PEA and was witnessed by EMS. The case was described as massive external bleeding, so fluid resuscitation was a priority over epinephrine administration during CPR. ALS. Ultrasound confirmed the absence of a heartbeat, so CPR was abandoned after 31 minutes although the rhythm was PEA. Six minutes later, the patient was noticed to be swallowing and had a detectable blood pressure (120/50). The ECG showed sinus rhythm 30/min and the temperature was 32.7°C. The patient was taken to hospital but died 88 minutes after ROSC had occurred. (3) 63y. M. Carbon monoxide poisoning. Unwitnessed arrest. When EMS arrived, initial rhythm was PEA. Full ALS including one shock. Resuscitation was abandoned after 12 minutes (rhythm PEA). Autoresuscitation (junctional rhythm 118/min) occurred after 3 minutes as shown by breathing, a palpable carotid pulse and capnography. BP 170/80. The patient was taken to hospital but died >26 hours later. (4) 91y. F. Witnessed collapse in a nursing home. Initial rhythm was PEA and remained so. BLS performed. An endotracheal tube was used for airway management. There was a significant amount of bleeding in the endotracheal tube (no further details given). Neither defibrillation nor epinephrine were used. Resuscitation was abandoned after 16 minutes (rhythm still PEA). Three minutes later, autoresuscitation occurred. The ECG showed sinus rhythm 45/min and there was breathing and a palpable carotid pulse. This lasted for 15 minutes after which, the patient died. (5) 61y. F. EMS-witnessed collapse due to aortic dissection. Initial ECG showed PEA. Full ALS. 3 doses of epinephrine but no defibrillation. Ultrasound confirmed the absence of a heartbeat, so CPR was abandoned after 18 minutes, although the rhythm was PEA. A norepinephrine infusion had been used during resuscitation and this was left running. Eight minutes later, breathing noticed but other movement. ECG showed a junctional rhythm (40/min). The patient died 3 minutes later.^18^
- 81y. M. Permanent pacemaker. Undergoing operation for endovascular repair of a ruptured thoracic aortic aneurysm with mediastinal bleeding. Initially, the procedure went fine but when an attempt was made to remove the femoral catheter, there was profound bleeding and an exploration revealed extensive damage to the external iliac artery. IV blood /fluids and epinephrine. Ventricular complexes widened, and asystole occurred soon after, despite persistent pacemaker action. The arterial pressure tracing confirmed no cardiac output. CPR. Resuscitation attempts were abandoned 25 minutes after the blood pressure first dropped (5 minutes after asystole occurred) because of the large vascular injury, persistent asystole, patient age and the underlying disease (mediastinal haemorrhage of the thoracic aortic aneurysm). At this point the ECG only showed pacemaker activity without intrinsic cardiac activity and the arterial pressure trace was flat. The ventilator was turned off and the endotracheal tube disconnected. Two minutes later, ventricular complexes associated with pacemaker spikes were noticed on the ECG. The blood pressure returned, and the patient’s pupils narrowed. The operation to repair the surgical injury was completed and the patient taken to ICU. He was transferred to his local hospital after two weeks and to home after that. He showed no neurological deficits. He subsequently died at home a few days later (no details).^19^
- 50y. M. Three-day history of abdominal pain. Previous total gastrectomy. Haematemesis. CT showed a dense lesion on the hepatic artery. Hypotensive. During oesophagoduodenoscopy, he had a very large haematemesis that led to haemorrhagic shock. Intubated. Massive transfusion (25 units packed red blood cells). Emergent angiography for diagnosis and treatment of massive upper GI bleeding. This showed a 1 cm pseudoaneurysm of the hepatic artery with massive extravasation. While attempting coil occlusion, the patient lost consciousness and had a cardiac arrest. ALS. There was no response to 30 minutes of resuscitation, so the decision was made to stop resuscitation and extubate the patient. At this time, the patient was asystolic and had no blood pressure. After 2 minutes of asystole and absent blood pressure, the ROSC occurred. He was reintubated, and the procedure was completed with the placement of a second coil which successfully stopped the bleeding from the common hepatic artery. The patient was taken to ICU where he made a full recovery. He initially had some short-term memory loss, but this was expected to resolve over time. He was discharged 11 days after the treatment and had no further bleeding. He died 8 months later from recurrent cancer.^20^
- 53y. M. Attempted suicide with shotgun. Underwent extensive surgery. ICU post-op. Multiple complications and needed pharmacological cardiac support. During a surgical procedure to repair an iatrogenically-damaged femoral artery, he arrested. Full ALS instituted. After 46 minutes resuscitation, he went into asystole, so resuscitation was abandoned. Two minutes later, normal sinus rhythm resumed. Spontaneous breathing never resumed and there was evidence of hypoxic brain damage. He remained alive for 34 days.^21^
- 36y. F. Lifelong asthmatic. Acute severe asthma unresponsive to standard medical treatment. Decision was taken to ventilate the patient. After intubation, hand ventilation was commenced at a rate of ≈14/min and estimated tidal volume of 6-700 ml. SpO_2_ initially increased but five minutes after intubation, the blood pressure was unrecordable although the carotid pulse was palpable. Two minutes later, the pulse was also not palpable. Full ALS over the next 15 minutes. Persistent PEA. No pneumothorax. 25 minutes after intubation, the situation was considered irretrievable and resuscitation attempts were abandoned. Ventilation was stopped. Three minutes later, the heart rate had increased to 115/min sinus rhythm and two minutes later, peripheral pulses were palpable. Ventilation was recommenced at a rate of fifteen breaths per minute and within fifteen seconds the carotid pulse was again impalpable. When the ventilation was reduced to 6-8/minute, with a short inspiratory phase and a prolonged expiratory phase, the pulse returned. The asthma gradually subsided but the patient had sustained severe hypoxic brain damage. By six months she was functioning independently at home with minimal persisting neurological deficit.^9^
- 89y. F. Significant co-morbidities including cardiac and respiratory problems. Fell and fractured her wrist and neck of femur. Bradycardia leading to asystolic cardiac arrest. ALS. Asystole persisted. Resuscitation abandoned after 18 minutes. Five minutes later, a heartbeat was noticed and two minutes after that, normal sinus rhythm resumed. Initial blood pressure was 80/50 mmHg. Seven hours later, she arrested again and died.^22^
- (Two paediatric cases reported). (1). 9m. F. Found apnoeic and pulseless. Bystander CPR. EMS arrived and found pulseless narrow-complex bradycardia 24/min. Full ALS care. Vascular access via the intraosseous route. ALS care continued in the ambulance. On arrival in the ED, the patient was asystolic. ALS care was continued. Ultrasound assessment was performed after 10 minutes and demonstrated cardiac standstill, so resuscitative efforts were stopped. Within 30 seconds of stopping CPR, she had a narrow-complex tachycardia and femoral pulses were palpable. The ultrasound scan was repeated and the heart could be seen beating weakly. Transferred to PICU but shown to have severe brain injury. Life-sustaining therapies were withdrawn several days later. (2). 3y. M. Medulloblastoma. Admitted to PICU with shock and neutropenia. Developed an acute abdomen. Intubated for respiratory distress. Sepsis. Did not respond to treatment. Peritoneal and pleural effusions drained. Minutes after the chest tubes were placed, he became bradycardic, which progressed rapidly to asystole. CPR and ventilation through the endotracheal tube. PaCO2 after 4 minutes of CPR was 44 mm Hg (down from 61 mm Hg just before the arrest). 25 minutes of full ALS and aggressive fluid resuscitation. He remained asystolic. Resuscitation efforts were discontinued. Within 1 minute of stopping CPR, he developed an organized narrow complex rhythm (rate of 66 bpm) accompanied by a palpable femoral pulse and a blood pressure of 77/27 mm Hg. Unfortunately, 40 minutes after ROSC, blood pressure fell again and was unresponsive to treatment. After discussions with the family, life-sustaining therapies were withdrawn.^23^
- 64y M. Acute exacerbation of chronic obstructive pulmonary disease. Deteriorated. Intubated to support breathing. Shortly after this, blood pressure fell to 60 systolic. Vasopressors. IV fluids. No response. PEA. ALS commenced. No obvious reversible factors. Resuscitation abandoned after 20 minutes (rhythm not stated). Fifteen minutes later, patient was noted to have spontaneous respirations, sinus tachycardia, and a systolic blood pressure of 60 mm Hg. Transferred to ICU but died with profound hypotension in an hour despite aggressive therapy. The authors of the paper felt that rapid ventilation had allowed air trapping to occur leading to hyperinflated lungs with consequent effects on the circulation.^24^
- 76y. M. Chronic lung disease on a mechanical ventilator in intensive care. Bradycardia progressing to cardiac arrest. CPR. Defibrillation. Progressed to asystole despite pharmacological attempts to treat various arrhythmias. After 30 minutes CPR, resuscitation was abandoned due to continuous asystole. Five minutes later, cardiac activity was noted on the ECG accompanied by a good blood pressure and attempts to breathe. Neurological assessment identified significant hypoxic brain damage and the patient died 24 hours later.^25^
- 44y. M. Collapsed in the street. Immediate bystander CPR followed by EMS. Emergency medical assistance arrived to facilitate advanced techniques. Full ALS. Mechanical CPR (LUCAS 2). Intubated and mechanically ventilated on 100% oxygen. Continuous CO_2_ monitoring and repeated evaluation by ultrasound and echocardiography. ECG showed intermittent VF which responded to defibrillation. After 80 minutes, there was persistent PEA 25/minute despite the administration of inotropes. Pupils were fixed and dilated. Ventricular akinesia was demonstrated. Around that time, there was a precipitous fall in end-tidal CO_2_. A team decision was taken to discontinue CPR. The ECG monitor was left running, which is current practice in Germany in the presence of persistent PEA. The ventilator was disconnected from the endotracheal tube, and the tube left in place. Five minutes after withdrawal of all supportive measures, audible breathing resumed. A bradycardic pulse became palpable (carotid) and QRS-complexes increased in frequency and appeared increasingly narrow. Capnometry showed a spontaneous increase of end-tidal CO_2_ to 30 mmHg. Focused cardiac ultrasound showed coordinated ventricular activity with reduced ventricular output. Catecholamine support was restarted. The patient was ventilated and targeted temperature management initiated. Shortly after he had a systolic blood pressure of 130 mmHg, an end-tidal CO_2_ of 35 mmHg and a peripheral SpO_2_ of 99 % on 50% oxygen. 0.5. The ECG became normal and the pupils started reacting. The authors felt that the rapid decline in end-tidal partial pressure of carbon dioxide supports the hypothesis of increased intrathoracic pressure. The patient was taken to hospital where he had further resuscitation. However, a CT scan showed severe neurological damage and so had palliative care only.^26^
- 87y. F. Asthma. Collapsed on the ward. BLS started immediately. ALS confirmed VF arrest. ETT. Defibrillation attempt. Asystole. Escalating doses of epinephrine totalling 10 mg. Fine VF resumed. A second shock caused ROSC. The pulse became weaker. Wide complexes (no mention of asystole). Second attempt at escalating dose of epinephrine (10 mg) failed. After 15 minutes ALS, resuscitation was abandoned. The ETT was removed and the patient was immediately noted to be gasping. Palpable pulse and blood pressure of ≈120. Aspirated gastric contents. Patient taken to ICU and ventilated. Opened her eyes spontaneously but no other responses. Died 12 days after original arrest.^27^
- 63y. F. Multiple severe medical problems including COPD and chronic renal failure and digoxin toxicity. Haemodialysis. VF arrest 50 minutes after starting dialysis. ALS. During CPR, it was noted that manual ventilation was difficult. Asystole occurred after two cycles of ALS in which VF was present. The asystole persisted for two minutes after which time, resuscitation was stopped after a total of 12 minutes based on no response to resuscitation and very poor prognosis from underlying disease. Three minutes later, while the ECG leads were still connected, ROSC occurred with palpable blood pressure. Patient had normal mental function but died 12 days later from sepsis. The authors accept that transient asystole can occur immediately after defibrillation.^5^
- 59y. F. Known ischaemic heart disease. Chest pain. Dyspnoea. Acute myocardial infarction with left ventricular failure. Witnessed PEA arrest. In-hospital BLS and ALS. After six loops of ALS (≈12 minutes) with 1 mg epinephrine administered in each loop, a 5mg dose of epinephrine was administered. Resuscitation terminated 3 minutes later when no response. The rhythm at TOR is not stated but might have still been PEA as there is no mention that the rhythm had changed. Ventilation stopped. Two minutes later, the patient was observed to be making respiratory efforts. ECG showed narrow complex tachycardia, initially with a good blood pressure. However, the patient deteriorated and died 30 minutes later. The author felt that restoration of the circulation might have been due to delayed delivery of epinephrine to the heart.^28^
- 84y. M. Previous history of major cardiovascular disease and mild dementia. Witnessed arrest. No bystander CPR but Mobile Emergency Care Unit arrived within four minutes. Full ALS care provided. Resuscitation was abandoned after 15 minutes of refractory VF. Five minutes after cessation of treatment, spontaneous, normal breathing recurred. The blood pressure was 120/84mmHg and the pulse regular. ECG, echocardiography and subsequent CT were unchanged from prior examinations. The patient was discharged on day 17, having reached his previous level of mental and cardiorespiratory capacity. After 1 year, the patient was doing well except for minor cognitive deficits.^29^
- 67y. M. Severe concurrent cardiovascular, respiratory, and renal disease. Witnessed arrest. Immediate bystander CPR. EMS on-site within 3 minutes. VF. ALS. 20 defibrillations delivered. 50 minutes after the arrest, he arrived at hospital still in VF. This changed to PEA after a further defibrillation. Resuscitation was abandoned 55 minutes after the arrest. The monitors were turned off. Two minutes later, slow agonal breathing was observed, and five minutes later a very faint central pulse was detected. Because of severe comorbidities, the decision was taken to just deliver palliative care. The patient regained consciousness and one hour later, he blinked and squeezed hands on command and three hours later he sat up in his bed and had some soup. However, he died 22 hours later.^30^
- 27y. M. Known heroin and cocaine user. Collapsed after injecting drugs intravenously. EMS called. He recovered consciousness after IV and IM naloxone and walked to the ambulance. However, his breathing rate decreased during transport and he had a cardiorespiratory arrest. BLS commenced. In hospital, he was in asystole and remained so despite 25 minutes of ALS. The decision to discontinue resuscitation was taken after 25 minutes of timed resuscitation when the patient was still asystolic and pulseless with no respiratory effort. The time of death was noted verbally. About 1 minute later, a rhythm was seen on the monitor and the radial pulse was palpable. The first systolic blood pressure recorded was 140. Assisted ventilation was restarted and the patient was transferred to ICU. Although he had complications following the arrest, he was discharged after 18 days having made a full recovery.^31^
- 21y. M. Duchenne muscular dystrophy with cardiomyopathy. ICU for respiratory problems. Asystolic arrest that did not respond to 30 minutes ALS. Five minutes after cessation of CPR, the ECG still showed asystole. However, 5 minutes later (10 minutes after cessation of resuscitation), normal sinus rhythm with a palpable pulse was noted, and the patient was reintubated. Sixty days after admission, the patient was discharged from hospital with complete neurological recovery.^32^
- 80y. M. History of cardiovascular disease. Admitted with severe pulmonary oedema. Shortly after, cardiac arrest occurred. Full ALS commenced. After 20 minutes, there was no pulse, spontaneous breathing, or cardiac activity and the pupils were dilated and unresponsive. CPR was discontinued. Five minutes later, a nurse noticed some breathing movements although the ECG monitor, which was still connected, showed asystole. CPR and ventilation were restarted for a few seconds after which, a pulse returned, and the heart was in sinus rhythm. ICU. Treated for cardiac failure secondary to myocardial infarction. A few hours later, the patient had fully regained consciousness. He was ventilated for 30 days because of a chest infection and was discharged home five days later, fully able to care for himself.^33^
- 83y. M. Ischaemic cardiomyopathy and atrial fibrillation. Admitted with increasing dyspnoea. Bradycardia (ECG monitor) led to a witnessed cardiac arrest. ALS and attempted external pacing failed. Blood gases showed a metabolic acidosis, advanced renal failure and hyperkalaemia (9.4 mmol/L). Appropriate treatment for hyperkalaemia was instituted. Internal pacing was also attempted but failed. After 60 minutes, there was still no response (ECG rhythm not stated) and resuscitation was abandoned. 7 minutes later, a pulse was detected by the pulse oximeter. The patient went on to make a full recovery 24 hours later.^6^
- 10y. F. Multiple congenital neurological conditions. Tracheostomy and home ventilation. At 03:30 the patient’s respiratory alarm sounded. Patient pale and unresponsive. EMS arrived. Asystole. ALS. PEA emerged. Transported to hospital. Further ALS. VT/VF treated with defibrillation. PEA returned. After 40 minutes, further resuscitation was stopped, and death declared at 4:17 a.m. At 4:19 a.m., the bedside nurse was removing the patient’s monitor leads when a palpable pulse was noticed. Blood pressure 79/46. Additional epinephrine followed by an infusion was administered, with increase in heart rate from 85 to 110 bpm and blood pressure 114/63 mm Hg. Patient transferred to ICU. Neurologic exam revealed fixed, dilated pupils and the absence of brain stem reflexes. After discussion with the patient’s mother, the decision was made to withdraw care.^34^
- 70y. M. Chronic renal failure. Developed a wide-complex bradycardia due to hyperkalaemia (potassium 8.5 mmol/L). This became pulseless (rate 25/min). Full ALS care. Calcium gluconate. Then pulseless VT. Dextrose and insulin, defibrillation and other drugs to attempt to terminate the abnormal rhythm. Ten minutes after CPR was started, PEA which degenerated to asystole. This persisted despite multiple attempts, including pacing, to re-establish a rhythm. Resuscitation was terminated. ROSC occurred 8 minutes later. Although technically a reversible cause, the authors have suggested this was autoresuscitation because there was a lag between the administration of appropriate treatment and the response. Discharged neurologically intact 3 weeks later.^7^
- (2 cases reported). (1). 66y. M. Brought into the Emergency Department in cardiac arrest following myocardial infarction. After 30 minutes resuscitation, still in refractory VF. CPR terminated but mechanical ventilator left running. Moments later, ROSC occurred without any further interventions. Patient made a good recovery but was left with a left hemiparesis. (2). 71y. M. Admitted with severe anaemia. Cardiac arrest on the ward. Team arrived and found the patient in VF. Repeated defibrillation attempts. 35 minutes later, the ECG showed asystole and resuscitation was abandoned. Moments later, the patient was noticed to be gasping and was in a normal rhythm with an effective cardiac output. Patient made a full recovery. The authors point out that transient asystole can occur after a defibrillation and may last for some time. Therefore, this is not a reason to terminated CPR at that stage.^35^
- 94y. F. Undergoing emergency laparotomy for perforated duodenal ulcer. Sudden haemorrhage (estimated 1500 ml). Transfused. At the end of the operation, QRS amplitude increased accompanied by bradycardia. Degenerated into PEA. CPR. ALS. PEA persisted (rate 35/min). Reversible causes were excluded. Resuscitation abandoned after 40 minutes. Ventilator disconnected but endotracheal tube left in place and ECG and arterial pressure monitor left attached. After 2 -3 minutes, the heart rhythm restarted spontaneously, and arterial pressure waves reappeared on the monitor. The patient progressed well for 72 hours, after which she developed septic shock and multi-organ failure and died 18 days later.^36^
- 11m. F. Complicated medical history including hypoxic encephalopathy, seizures, and cardiomyopathy with atrioventricular block (types I and II). Admitted to hospital for investigations. Noticed to have a baseline bradycardia (for a child) of 50-70/min with occasional drops to 20/min triggered by a nasogastric tube. The heart rate returned to normal when the tube was removed. The next day, the tube was replaced to allow medication to be given. The patient’s heart-rate monitor alarmed and she was found to in asystole, possibly triggered by a seizure. BLS was effective. While being transferred to PICU, another seizure again caused slowing of the heart rate slowed leading on to asystole. In PICU, the child was intubated and put on a ventilator. She continued to have recurrent episodes of haemodynamic instability associated with various arrhythmias. Brief periods of CPR were required on several occasions to re-establish a perfusing rhythm. This repeatedly degraded into a bradyarrhythmia or non-perfusing tachyarrhythmia. Despite several different cardiac drugs, the bradycardia progressed (10-20/min). Because of the poor prognosis, CPR was not instituted, and the patient became asystolic for >2 minutes, after which point, she was declared dead. The ventilator was left running (reason not stated). Fifteen minutes later, while removing the endotracheal tube, the patient was noted to have a gag reflex. Oxygen was administered and the heart rate monitor, which had been disconnected after diagnosis of death, showed 40/min. She started to cry and opened her eyes spontaneously. Full care was re-instituted. She remained in PICU for a month and was gradually weaned off all cardiac pharmacological support. She died 6 weeks later from progressive cardiomyopathy.^37^
- 65y. M. Found unconscious at home. Brought into hospital. In the ED, cardiac arrest confirmed. ALS. Intubated. VF/VT at times. After 55 minutes, persistent asystole and the patient was pronounced dead. Resuscitation was stopped, and the monitors turned off. 40 minutes after resuscitation had been stopped, the patient was noticed to be breathing. Reintubated. Good blood pressure. ECG confirmed myocardial infarction. Post resuscitation, the patient was maintained on mechanical ventilation for 13 days in the ICU. He was gradually weaned off inotropes. He developed acute kidney injury requiring renal replacement therapy. The patient’s GCS level remained very low (≈3) always and CT brain imaging, which was initially normal on Day 1, subsequently showed features of hypoxic ischaemic encephalopathy and bleeding. The patient deteriorated and was pronounced dead on Day 13 after admission.^38^
- 63y. M. Found in his unheated house. Speech disorder and left hemiparesis. GCS 14. BP 115/71. Walked to the ambulance and had a VF arrest. Full ALS including large doses of epinephrine (22 mg). Resuscitation terminated after 70 minutes due to refractory asystole. ROSC noticed to have occurred 10 minutes later. In hospital, the patient was noted to have severe hypothermia (23°C). The patient’s temperature was unknown at the time of the arrest. Full recovery.^8^
- 35y. Normothermic cardiac arrest following myocardial infarction. Full ALS including 60 mg epinephrine. Resuscitation terminated after 88 minutes CPR (rhythm not stated). “Shortly afterwards”, ROSC occurred, and the patient was transferred to ICU. Fifty minutes later, a further arrest occurred. CPR continued for a further 30 minutes but unsuccessful.^39^
- 83y. M. COPD with respiratory difficulties. Concurrent medical problems included cirrhosis with portal hypertension. Cardiac arrest 10 years previously. Weak signs of life when EMS arrived. Intubation triggered severe bronchospasm followed by cardiac arrest. Sequence of cardiac rhythms including VF and pulseless VT. Full ALS care. After 90 minutes, refractory asystole so resuscitation abandoned. Ten minutes later, with the patient now extubated, ROSC occurred. The patient was re-intubated and transferred to hospital. He died 12 days later, in hospital, of pneumonia due to aspiration of microorganisms after the first cardiac arrest.^40^
- 85y. F. Acute onset of dyspnoea. Previous history of coronary artery disease and cardiac failure. When EMS arrived, she was gasping and had a GCS 3 and non-reacting pupils. Immediate BLS. Initial ECG showed broad complex bradycardia (31/min) with no palpable pulse. This changed to VT which changed to VF. ALS. Intermittent periods of asystole which became continuous after the third shock. Resuscitation was terminated 14 minutes after this because of persistent asystole. Two minutes later, whilst the chest was being auscultated, breathing movements were noticed followed by a palpable femoral pulse. ECG showed a rate of 90/minute. Ten minutes later, the complexes again became wide and asystole resumed. The patient died 25 minutes after signs of life had returned.^41^
- 62y. M. History of complete heart block and implanted pacemaker. Soon after admission to ICU for sudden loss of consciousness, the patient had a VF arrest. Full ALS care including multiple doses of epinephrine and defibrillation attempts. The predominant rhythm was VF with uncaptured pacemaker spikes. This degenerated into an idioventricular rhythm with uncaptured pacemaker spikes. Resuscitation was terminated after 10 minutes of sustained pulseless idioventricular rhythm (40 minutes total resuscitation time). Ventilation was continued until the pacemaker could be deactivated. Five minutes after termination of resuscitation, a spontaneous circulation resumed 55-75/min. Pacemaker spikes were seen without capture occurring. This lasted for 34 minutes at which point, circulation ceased. The pacemaker was deactivated 2 hours later without further resumption of cardiac activity having occurred.^42^
- 55y. M. Obese. Witnessed collapse. No bystander CPR. EMS arrived in five minutes. BLS. Full ALS when the doctor arrived. Multiple defibrillation attempts and epinephrine. Sodium bicarbonate was also administered. Mechanical CPR. Transthoracic external pacing was attempted at 17 minutes after the start of CPR when the rhythm had changed to asystole with occasional broad complex beats (≈22 minutes after arrest). Resuscitation attempts were abandoned 35 minutes after the witnessed collapse (30 minutes after the start of CPR). The ECG monitor was left running. Seven minutes after resuscitation was discontinued, ROSC occurred and there was a palpable radial pulse. The dilated pupils narrowed. The patient was admitted to ICU where it was found that he had significant co-morbidity including probable renal impairment. The latter led the authors to speculate that this might have been a hyperkalaemic arrest and the bicarbonate had been beneficial. A serum potassium after ROSC was 5.7 mmol/l. An ECG and blood tests did not show that a myocardial infarction had occurred. The patient died three days later from profound cerebral oedema.^43^
- (2 cases reported). (1) 63y. F. History of paroxysmal atrial fibrillation, COPD, Crohn’s disease, and hypothyroidism caused by amiodarone. Long-term medication for these conditions. History of increasing dyspnoea not related to her chronic chest problems and R heart failure. EMS called. SpO2 82% on oxygen. Sudden bradycardia and loss of consciousness. No response to atropine or isoproterenol. Rapid deterioration to asystole. CPR and ALS but no response. Resuscitation abandoned after 40 minutes because of persistent asystole. The family were informed. 10 minutes later, a carotid pulse was noticed. BP 105/60. ECG sinus rhythm 50/min. No further treatment was given, and the rhythm degenerated back to asystole a few minutes later and the patient died. (2) 78y. F. Five minutes chest pain followed by witnessed cardiac arrest. Previous medical problems included COPD, renal carcinoma, and chronic renal failure. No bystander CPR. EMS arrived 15 minutes later. ALS. Because of the history of chronic renal failure, calcium chloride was also given in case hyperkalaemia was present. 41 minutes after resuscitation started, the patient was in asystole and the decision was taken to terminate resuscitation. Whilst the family were being informed, a nurse noticed breathing movements two minutes after extubation. These increased and a pulse became palpable. This continued for about 15 minutes and then faded over five minutes. Resuscitation attempts were not resumed.^44^
- 51y. M. Collapsed at an ophthalmology clinic due to myocardial infarction. Soon went on to have a cardiac arrest. ALS. CPR continued for 60 minutes during which the patient received 26 defibrillations, 39 mg epinephrine and other ALS drugs. Resuscitation was terminated at 61 minutes for persistent asystole. 3-4 minutes later, breathing movements were noticed and a carotid pulse was palpable. The patient was transferred to ICU and continued to improve. After a period in rehabilitation, the patient was discharged home after 15 days having made a good recovery. 9 months later, he had residual amnesia and numbness in the R arm but was otherwise well.^45^
- 78y. M. Found on floor in his apartment. He had probably lying there already for >24 hours. He was still breathing and was initially rousable. EMS called. When they arrived, there was possibly a weak pulse and GCS 3. Ventilated using a self-inflating bag. Blood pressure unrecordable and one pupil dilated (the other eye had been enucleated). ECG showed asystole. ALS commenced. ECG remained predominantly asystole with occasional wide-complex bradycardia (agonal rhythm). Defibrillation was attempted. Epinephrine. Resuscitation was abandoned 31 minutes after the start for persistent asystole. Death was declared. Three hours 40 min later, the patient had signs of life noticed by the undertaker. EMS called and found sinus rhythm 50/min, BP 90 mmHg systolic, respiratory rate 10/min, SpO2 84%. No response (GCS 3). Patient taken to hospital but died 19 hours after the initial arrest.^46^
- 85y. M. Previous history of aortic valve replacement and permanent pacemaker for bradyarrhythmia. Found collapsed at home. Initially no signs of life. Resuscitation started, and the patient stabilised and taken to hospital where he was found do have a ruptured abdominal aortic aneurysm. The patient was taken for operation where he was also found to have infarcted bowel. Anaesthesia included inotropic support. During mobilisation of the bowel, the blood pressure fell. Suddenly there was a marked broadening of the ventricular complexes and the heart stopped capturing the pacemaker pulses. Arterial blood pressure was no longer measurable at this time and the end-tidal carbon dioxide fell. Because of the history and clinical findings, the decision was taken not to start CPR. Ventilation was disconnected while leaving the endotracheal tube in place, catecholamine therapy stopped, and abdomen closure started. The monitors were left running which showed pacemaker pulses only. Six minutes after the cardiac arrest, ROSC occurred with measurable arterial blood pressure (100/60 mmHg) and peripheral oxygen saturation of 92%. Blood results were not significantly abnormal. The operation resumed and afterwards, the patient was taken to ICU. The patient did open his eyes but had features of a vegetative state. Because of this and the rest of the history, treatment was withdrawn, and the patient died two days later.^47^
- 69y. M. Alzheimer's. Brought to hospital by his relatives suffering from a respiratory complaint. They arrived at the Emergency Room at 10:20. Cardiac arrest on arrival. ALS. At 11:00 resuscitation attempts were abandoned due to refractory asystole. The patient was taken to the morgue. At ≈14:00, the relatives were visiting the body and noticed he was breathing. The medical team attended and confirmed that the patient was alive. Admitted to ICU at 14:30. The patient remained unconscious. Atrial fibrillation 82/min, blood pressure 75/55 mmHg. Bronchopneumonia. Mechanical ventilation. Inotropes. CT showed hypoxic brain damage. The patient slowly deteriorated and arrested again on 10^th^ day. Resuscitation attempts were unsuccessful.^48^
- 54y. F. Out-of-hospital cardiac arrest. BLS on scene. Admitted to hospital 30 minutes later. ALS in hospital. After 20 minutes resuscitation in hospital, PEA thought to be due to profound acidosis (pH 6.54; pCO_2_ 11.2; Base Excess -36.7). This situation was thought not to be survivable, so resuscitation stopped. A few seconds later, pulse and breathing restarted. Severe hypotension persisted (mean arterial pressure <45 mm HG) and was treated with vasopressors. After >210 mins, the haemodynamic conditions spontaneously improved and the patient woke up without any signs of hypoxic encephalopathy. The patient had a very stormy and protracted post-arrest stay in ICU and was eventually discharged from hospital after 93 days having made a full recovery.^49^
- 46y. m. Diabetic. Witnessed out-of-hospital VF arrest. Immediate ALS. Resuscitation efforts were continued for 30 minutes. ROSC. Patient taken to hospital. On arrival, ECG showed a junctional rhythm that changed to asystole soon after. ALS resumed for 30 minutes. Bedside ultrasound revealed asystole. The patient was pronounced dead. One hour later, the patient was seen to move spontaneously. ROSC had occurred (pulse 92/min; BP 115/80). The patient was awake and obeyed simple commands. ECG showed acute myocardial infarction. PCI with stents. Chest X ray showed right-sided pneumothorax occupying 50% of the lung volume with multiple rib fractures. A chest tube thoracostomy. Despite maximal ventilation, the patient’s oxygenation continued to decline, and cardiogenic shock developed. Despite aggressive resuscitation, the patient’s cardiopulmonary status continued to decline until he became asystolic. Resuscitation was continued for 45 minutes but asystole persisted, and the patient was pronounced dead.^50^
- 18m. M. Unexplained collapse at home. CPR started at the scene and continued by paramedic and later, hospital staff. Standard ALS management. After 10 cycles of CPR in hospital with 5 doses of intravenous epinephrine, CPR was ceased with the agreement of the entire resuscitation team. Echocardiography was not performed during the resuscitation. There was no sign of life, no palpable pulse (carotid or femoral), no response to painful stimulus, and no pupillary response to light. After stopping resuscitation, the medical team left the room, leaving the family and staff with the patient. The monitors were turned off. The parents noted a small movement approximately 6 minutes later. A further movement was noted prompting a reassessment at which time a pulse was palpable. The team was recalled, resuscitation recommenced with the patient in sinus at a rate of 70 beats per minute. The patient was transferred to the paediatric intensive care unit. The patient survived, but with significant cerebral impairment that had not improved by 1 year.^51^
- 83y. F. Admitted to hospital for investigation of tiredness. Hypertension and atrial fibrillation. VF arrest. ALS. Resuscitation discontinued after 15 minutes due to asystole and poor general condition of the patient. There were no signs of life. 10 minutes later, spontaneous breathing was noted. ECG showed sinus rhythm. The patient did not recover consciousness. Two hours later, she had a further cardiac arrest. Resuscitation was not attempted.^52^
- 25y. F. Laparoscopic cholecystectomy. During insufflation of CO_2_, end tidal CO_2_ concentration (EtCO_2_) started to gradually fall. Heart rate fell from 90 to 50 and SpO_2_ fell from 100% to 70%. Insufflation stopped. NIBP 56/28. Manual ventilation on 100% oxygen. Inhalational anaesthetic stopped. VF arrest. CO_2_ embolism was suspected. ALS (defibrillation; 4 x 1 mg epinephrine). After 40 minutes, resuscitation was abandoned (cardiac rhythm not stated). 5-7 minutes after discontinuation of CPR, the ECG on monitor showed an idioventricular rhythm (no palpable pulse) and 5 minutes later, SR at a rate of 96/minutes. The heart sounds were confirmed, and a low volume carotid pulse was palpable. NIBP 60/32 mmHg. IV fluids and inotropes. Pulse oximeter reading appeared on the monitor. Blood pressure continued 70-80 mmHg systolic. Patient transferred to ICU for mechanical ventilation. Inotropes continued. GCS 3. Arterial blood gas analysis showed severe metabolic acidosis and hypoxia. Therapeutic hypothermia was induced. After 4 hours the patient again arrested and could not be revived.^53^
- 66y. M. Leaking abdominal aortic aneurysm. Emergency operation. Long-standing chronic illness. Uneventful induction of anaesthesia. Operation started. The aorta was cross clamped. The blood pressure rose and a few minutes later, VT degenerated to VF. ALS for 17 minutes. However, rhythm degenerated to asystole. End-tidal CO2 fell, and pupils were widely dilated. Resuscitation was abandoned 18 minutes after the transition to VT. Medication, ventilation and monitoring were stopped. The surgeon stayed in the OR to demonstrate the anatomy to medical students. Ten minutes after abandoning resuscitation and declaring death, the surgeon announced he could feel a pulse in the proximal aorta. Ventilation recommenced. ECG showed SR, 90/min. Blood pressure 90. The operation resumed although the expected neurological outcome was bad. Ventilated on ICU post-op. Discharged home 13 days later. Full recovery.^54^
- 67y. F. Undergoing an endovascular operation for a thoracic aortic aneurysm. During the procedure, she had a VF arrest. ALS, including several doses of epinephrine and several defibrillations. The patient responded transiently but then re-arrested several times ultimately ending in refractory asystole. Five minutes later, a regular rhythm was noted on the ECG monitor, which had not been removed. The pulse was palpable, and the systolic blood pressure was 90. Therapy was resumed. The operation was abandoned, and emergency coronary angiography showed the cause of the arrest was a myocardial infarction. The patient underwent successful stenting of the affected vessel. By day 3, she had a normal pulse and blood pressure and was mentally normal. Unfortunately, on day 9, she died from complications of a pulmonary embolus.^55^
- 75y. M. Chronic cardiac failure. Possible acute rupture of the pulmonary artery during an investigative procedure. Cardiac arrest whilst being prepared for surgery. No response to ALS measures and the patient became asystolic. Resuscitation was terminated after 23 minutes. Five minutes later, he was noted to have a palpable femoral pulse. ECG showed sinus rhythm. CPR was resumed, and the patient taken to the intensive care unit. Though haemodynamic stability was obtained, no significant neurologic recovery was observed. All support was discontinued several days after the event and the patient died.^56^
- 66y. M. Chest pain. Prehospital VF arrest. Standard ALS management. One transient episode of ROSC followed by refractory VF. Arrived at hospital 18 minutes after the arrest. The patient remained unresponsive to further pharmacological attempts to terminate the VF. He was obviously deteriorating so resuscitation was abandoned 45 minutes after the original arrest and the relatives informed. The patient was noted to have developed PEA but from the report, this wasn’t acted on. He then started to take some breaths, though still had no palpable pulse. Over the next five minutes, the respiratory rate started to rise and shortly after, a carotid pulse became palpable. A 12-lead ECG confirmed a myocardial infarction. He was treated by the cardiologists (stent) and taken to intensive care. He was discharged from hospital nine days later having made a full recovery.^57^

**Case reports of autoresuscitation when outcome not stated**

- 93y. F. Septic shock and renal failure secondary to superior mesenteric embolism. Cardiac arrest developed 15 min after induction of anaesthesia. CPR started but was stopped after 6 min due to asystole despite a norepinephrine infusion in a severely ill 93-year-old. Ventilation was stopped. Five minutes after she was announced dead, cardiac activity was noticed on the monitor, which was still connected. The circuit was reconnected, and mechanical ventilation was started again. The authors felt that the cause of the cardiac arrest was positive pressure ventilation in the presence of decompensated septic shock. Terminating resuscitation efforts led to cessation of positive pressure ventilation and resumption of cardiovascular activity.^58^
- 40y. M. Presented at the ED with cardiac chest pain. VF arrest on route to the PCI centre. ALS. Refractory VF. Resuscitation attempts continued for 45 minutes by which time, the rhythm had deteriorated to asystole. Resuscitation was discontinued. The patient was extubated but continued to make agonal breaths (not stated how long this continued). 30 minutes later, the patient was making good respiratory efforts and started moving and localising to pain. Thrombolysis, re-intubation, and admission to ICU. The final outcome is not discussed in the report.^59^

**Case reports of autoresuscitation when no resuscitation was attempted**

- 52y. F. Attempted suicide by drug overdose. No signs of life when the EMS arrived at 10:00. An ECG was not done. 21 minutes later, the undertaker was attending to the body when he noticed breathing movements. The patient was immediately intubated and taken to hospital where she made a full recovery, other than femoral nerve palsy, probably related to the position she was lying in for a prolonged period.^60^
- 97y. M. Significant comorbidity. Atrial fibrillation with ventricular ectopics changed to VT, VF and then asystole for 9 seconds. The patient had a DNAR order, so no resuscitation was attempted. ROSC occurred spontaneously, and he woke up 2 minutes later. He subsequently died 20 hours later from his other illnesses. The ECG changes were captured and are displayed in the case report. It clearly shows asystole with resumption of a cardiac rhythm.^61^

**Case report of autoresuscitation where PEA was caused by excessive ventilation and ROSC occurred after cessation of ventilation**

- The authors wrote a letter about the importance of auto-PEEP due to excessive ventilation can cause PEA. In a review of PEA arrests at their institution, they reported three cases in which auto-PEEP is thought to have caused PEA and there was unexpected ROSC after discontinuation of ventilation because of the termination of resuscitative efforts. They did not provide any further information about these cases.^62^

**Case reports of autoresuscitation following failed withdrawal of life-sustaining therapy where details are available**

- (2 cases reported) (1). Age 40-50. M. Extensive traumatic spinal, chest, abdominal and pelvic injuries. Withdrawal of life-sustaining therapy (WLST) prior to possible organ donation after 12 days in ICU. At five minutes and 47 seconds, ECG and arterial pulsation ceased. One minute and 42 seconds later, ECG and arterial pulsation returned for a further one minute and 48 seconds before permanently stopping. (2) 57y. F. End-stage pulmonary fibrosis who had been ventilated in ICU for 21 days. WLST prior to possible organ donation. Six minutes later, transducer signal alarms indicated a loss of invasive arterial monitoring due to equipment failure. After this no arterial waveform analysis could be obtained. ECG activity ceased at eight minutes. After an absence of three minutes, ECG activity returned for three minutes before permanently stopping.^4^
- Aggregated data were presented for four cases where arterial blood pressure resumed following cessation of life-sustaining measures. The longest period of cessation of arterial blood pressure before resumption was 89 seconds. The duration of resumed activity ranged from 1 to 172 seconds. Measurable values for systolic and diastolic pressures during resumption were recorded for one adult subject and ranged from 12-27 mm Hg for systolic and from 11-15 mm Hg for diastolic.^63^

**Reports indicating that autoresuscitation occurred, but no patient data is available**

- Five cases of autoresuscitation were reported to the UK National Reporting and Learning System between 2009 and 2011 where the patient’s family was prematurely informed of the patient’s death after CPR efforts were stopped but the patient survived for a few more hours.^64^ As full clinical data is not available, these cases have not been included in the list above. Through clinical contacts, the National Patient Safety Agency (NPSA) is aware of five additional cases.^65^ Importantly, in these incidents, the problems appear to have arisen not because a five-minute observation period is too short but because staff wanted to break the news of the patient’s death to the patient’s family as soon as possible, and therefore spoke to the family in advance of carrying out formal procedures for confirming death.
- Five cases of autoresuscitation were reported in a review published in 1991.^66^ Unfortunately, the full text is not available, however, the English abstract indicates the nature of what happened. “*In Norway, death is defined as total and irreversible damage of the whole central nervous system. This means that the time of death is some minutes after circulatory arrest. The time lapse may vary from about five minutes up to 45 minutes, depending on the temperature in the brain when the circulation ceased. Five cases of spontaneous circulation after cessation of resuscitation are described. All the patients had asystole, diagnosed on ECG by anaesthesiologists. The resuscitation had lasted for 30 minutes when the crew of the ambulance, which included a doctor, gave up. Two of the patients left the hospital alive, three died after some hours. One of the patients had no cerebral sequelae, the other developed dementia. Circulation may also start spontaneously after the doctor has diagnosed circulatory arrest in patients suffering from suffocation and exsanguination”.*
- In a retrospective study of non-heart-beating organ donors, three of 31 patients who were being transported to a transplant centre for kidney donation on mechanical CPR regained spontaneous circulation. One made a full recovery (no further details published).^67^

**References**

[1] Hornby K, Hornby L, Shemie SD. A systematic review of autoresuscitation after cardiac arrest. Crit Care Med. 2010;38:1246-53.

[2] Hornby L, Dhanani S, Shemie SD. Update of a Systematic Review of Autoresuscitation After Cardiac Arrest. Crit Care Med. 2018;46:e268-e72.

[3] Peña SB, Aedo IF, Palomino SL. Spontaneous return of circulation after termination of cardiopulmonary resuscitation maneuvers: a systematic review of cases of Lazarus phenomenon. Emergencias 2014;26:307-16.

[4] Yong SA, D'Souza S, Philpot S, Pilcher DV. The Alfred Hospital experience of resumption of cardiac activity after withdrawal of life-sustaining therapy. Anaesth Intensive Care. 2016;44:605-6.

[5] Al-Ansari MA, Abouchaleh NM, Hijazi MH. Return of spontaneous circulation after cessation of cardiopulmonary resuscitation in a case of digoxin overdosage. Clin Intensive Care 2005;16:179-81.

[6] Torbado A, Pellejero S, Uriz J, Pavon A, Salvador M. No neurological impairment after a case of Lazarus phenomenon. Eur J Anaesthesiol. 2006;23:214 (A829).

[7] Quick G, Bastani B. Prolonged asystolic hyperkalemic cardiac arrest with no neurologic sequelae. Ann Emerg Med. 1994;24:305-11.

[8] Pasquier M, de Riedmatten M, Paal P. Autoresuscitation in Accidental Hypothermia. Am J Med. 2018;131:e367-e8.

[9] Rosengarten PL, Tuxen DV, Dziukas L, Scheinkestel C, Merrett K, Bowes G. Circulatory arrest induced by intermittent positive pressure ventilation in a patient with severe asthma. Anaesth Intensive Care. 1991;19:118-21.

[10] Meeker JW, Kelkar AH, Loc BL, Lynch TJ. A Case Report of Delayed Return of Spontaneous Circulation: Lazarus Phenomenon. Am J Med. 2016;129:e343-e4.

[11] Linko K, Honkavaara P, Salmenpera M. Recovery after discontinued cardiopulmonary resuscitation. Lancet. 1982;1:106-7.

[12] Maleck WH, Piper SN, Triem J, Boldt J, Zittel FU. Unexpected return of spontaneous circulation after cessation of resuscitation (Lazarus phenomenon). Resuscitation. 1998;39:125-8.

[13] Kamarainen A, Virkkunen I, Holopainen L, Erkkila EP, Yli-Hankala A, Tenhunen J. Spontaneous defibrillation after cessation of resuscitation in out-of-hospital cardiac arrest: a case of Lazarus phenomenon. Resuscitation. 2007;75:543-6.

[14] Maeda H, Fujita MQ, Zhu BL, Yukioka H, Shindo M, Quan L, et al. Death following spontaneous recovery from cardiopulmonary arrest in a hospital mortuary: 'Lazarus phenomenon' in a case of alleged medical negligence. Forensic Sci Int. 2002;127:82-7.

[15] De Salvia A, Guardo A, Orrico M, De Leo D. A new case of Lazarus phenomenon? Forensic Sci Int. 2004;146 Suppl:S13-5.

[16] Puschel K, Lach H, Wirtz S, Moecke HP. Ein weiterer Fall von “Lazarus-Phänomen”? . Notfall Rettungsmedizin. 2005;8:528-32.

[17] Backer H, Kyburz A, Bosshard A, Babst R, Beeres FJP. Dead or dying? Pulseless electrical activity during trauma resuscitation. Br J Anaesth. 2017;118:809.

[18] Kuisma M, Salo A, Puolakka J, Nurmi J, Kirves H, Vayrynen T, et al. Delayed return of spontaneous circulation (the Lazarus phenomenon) after cessation of out-of-hospital cardiopulmonary resuscitation. Resuscitation. 2017;118:107-11.

[19] Duck MH, Paul M, Wixforth J, Kammerer H. [The Lazarus phenomenon. Spontaneous return of circulation after unsuccessful intraoperative resuscitation in a patient with a pacemaker]. Anaesthesist. 2003;52:413-8.

[20] Wilseck Z, Cho K. Spontaneous circulation return after termination of resuscitation efforts for cardiac arrest following embolization of a ruptured common hepatic artery pseudoaneurysm. Gastrointes Interv 2015;4:55-7.

[21] Ekmektzoglou KA, Koudouna E, Bassiakou E, Stroumpoulis K, Clouva-Molyvdas P, Troupis G, et al. An intraoperative case of spontaneous restoration of circulation from asystole: a case of lazarus phenomenon. Case Rep Emerg Med. 2012;2012:380905.

[22] Huang Y, Kim S, Dharia A, Shalshin A, Dauer J. Delayed recovery of spontaneous circulation following cessation of cardiopulmonary resuscitation in an older patient: a case report. J Med Case Rep. 2013;7:65.

[23] Duff JP, Joffe AR, Sevcik W, deCaen A. Autoresuscitation after pediatric cardiac arrest: is hyperventilation a cause? Pediatr Emerg Care. 2011;27:208-9.

[24] Rogers PL, Schlichtig R, Miro A, Pinsky M. Auto-PEEP during CPR. An "occult" cause of electromechanical dissociation? Chest. 1991;99:492-3.

[25] MacGillivray RG. Spontaneous recovery after discontinuation of cardiopulmonary resuscitation. Anesthesiology. 1999;91:585-6.

[26] Hagmann H, Oelmann K, Stangl R, Michels G. Is increased positive end-expiratory pressure the culprit? Autoresuscitation in a 44-year-old man after prolonged cardiopulmonary resuscitation: a case report. J Med Case Rep. 2016;10:364.

[27] Martens P, Vandekerckhove Y, Mullie A. Restoration of spontaneous circulation after cessation of cardiopulmonary resuscitation. Lancet. 1993;341:841.

[28] Bradbury N. Lazarus phenomenon: another case? Resuscitation. 1999;41:87.

[29] Krarup NH, Kaltoft A, Lenler-Petersen P. Risen from the dead: a case of the Lazarus phenomenon-with considerations on the termination of treatment following cardiac arrest in a prehospital setting. Resuscitation. 2010;81:1598-9.

[30] Hannig KE, Hauritz RW, Grove EL. Autoresuscitation: A Case and Discussion of the Lazarus Phenomenon. Case Rep Med. 2015;2015:724174.

[31] Walker A, McClelland H, Brenchley J. The Lazarus phenomenon following recreational drug use. Emerg Med J. 2001;18:74-5.

[32] Adanali M, Güvenç TS, Kale MY. Lazarus phenomenon in a patient with Duchenne muscular dystrophy and dilated cardiomyopathy. J Acute Med. 2014;4:99-102.

[33] Letellier N, Coulomb F, Lebec C, Brunet JM. Recovery after discontinued cardiopulmonary resuscitation. Lancet. 1982;1:1019.

[34] Cummings BM, Noviski N. Autoresuscitation in a child: The young Lazarus. Resuscitation. 2011;82:134.

[35] Gomes E, Araujo R, Abrunhosa R. Two successful cases of spontaneous recovery after cessation of CPR. Resuscitation. 1996;31.

[36] Garcia JLC, Gonzalez-Latorre MV, Fernandez AN. El fenómeno de Lázaro: Reanimación espontánea (Spanish). Revista Espanola de Anestesiologia y Reanimacion 2004;51:390-94.

[37] Tretter JT, Radunsky GS, Rogers DJ, Daugherty LE. A pediatric case of autoresuscitation. Pediatr Emerg Care. 2015;31:138-9.

[38] Low DW, Looi I, Manocha AB, Ang HA, Nagalingam M, Ayop NA, et al. Rising from the dead! Med J Malaysia. 2012;67:538-9.

[39] Mutzbauer TS, Stahl W, Lindner KH. Compression-decompression (ACD)-CPR. Prehosp Disaster Med. 1997;12:S21.

[40] Aguado OR, Portilla FJS, Ruiz JPN. Lazarus phenomenon in an out-of-hospital emergency attended by a home emergency service. Emergencias. 2011;23:43-6.

[41] Tiesmeier J, Brandt O, Emmerich M. Unerwartete Lebenszeichen nach erfolgloser Reanimation (German). MMW Fortschr Med. 2010;152:33-6.

[42] Thong SY, Ng SY. Case report – Lazarus syndrome after prolonged resuscitation. Journal of Anesthesiology and Clinical Science: Herbert Publications Ltd; 2013.

[43] Voelckel W, Kroesen G. Unexpected return of cardiac action after termination of cardiopulmonary resuscitation. Resuscitation. 1996;32:27-9.

[44] Vaux J, Reveaux F, Hauter A, Chollet-Xemard C, Marty J. Le phénomène de Lazare (French). Ann Fr Med Urgence. 2013;3:182-83.

[45] Vorotyntsev SI, Goldovsky BM, Potalov SA, Serikov KV, Serbin VG, Nikolayenko AM, et al. The Phenomenon of Lazarus – Autoresuscitation after cardiac arrest (Russian). Emergency Medicine 2011.

[46] Monticelli F, Bauer N, Meyer HJ. Lazarus-Phänomen · Aktueller Stand der Reanimation und Fragen an den medizinischen Gutachter (German). Anaesthesist. 2006;16.

[47] Wiese CHR, Stojanovic T, Klockgether-Radke A, Bartels U, Schmitto JD, Quintel M, et al. Intraoperatives „Lazarus-Phänomen“? Spontane Kreislaufstabilisierung bei einem Patienten mit Herzschrittmacher (German). Anaesthesist. 2007;56:1231-36.

[48] Güven AT, Petridis G, Özkal SS, Kalfoglu EA. Lazarus Phenomenon in Medicolegal Perspective: A case report (Turkish). Adli Tıp Bülteni. 2017;22:224-27.

[49] Fumeaux T, Borgeat A, Cuenoud PF, Erard A, de Werra P. Survival after cardiac arrest and severe acidosis (pH = 6.54). Intensive Care Med. 1997;23:594.

[50] Jasra S, Majumdar S, Rajagopalan B, Fernandez S. Lazarus syndrome. A rare case of auto-resuscitation. American College of Physicians Annual Scientific Meeting – poster competition 2015.

[51] Mullen S, Roberts Z, Tuthill D, Owens L, Te Water Naude J, Maguire S. Lazarus Syndrome - Challenges Created by Pediatric Autoresuscitation. Pediatr Emerg Care. 2018.

[52] Alves S, Campos M, Reis G. Lazarus syndrome in the emergency room: A case report. Resuscitation. 2010;84:e151.

[53] Sukhyanti K, ShriKrishan C, Anu K, Ashish D. Lazarus phenomenon revisited: a case of delayed return of spontaneous circulation after carbon dioxide embolism under laparoscopic cholecystectomy. Anaes Pain Int Care 2012.

[54] Ben-David B, Stonebraker VC, Hershman R, Frost CL, Williams HK. Survival after failed intraoperative resuscitation: a case of "Lazarus syndrome". Anesth Analg. 2001;92:690-2.

[55] Frolich MA. Spontaneous recovery after discontinuation of intraoperative cardiopulmonary resuscitation: case report. Anesthesiology. 1998;89:1252-3.

[56] Bray JG, Jr. The Lazarus phenomenon revisited. Anesthesiology. 1993;78:991.

[57] Spowage-Delaney B, Edmunds CT, Cooper JG. The Lazarus phenomenon: spontaneous cardioversion after termination of resuscitation in a Scottish hospital. BMJ Case Rep. 2017;2017.

[58] Abdullah RS. Restoration of circulation after cessation of positive pressure ventilation in a case of "Lazarus syndrome". Anesth Analg. 2001;93:241.

[59] Mustamam AHM, Jailani AFMAK, Z’aba N, Balakrishnan SL, Ali NM. Arise from the dead – case report. Malaysian J Emerg Med. 2016;1:49.

[60] Klein A, Lach H, Püschel K. Ein weiterer Fall von „Scheintod” – Diskussion über Richtlinien zur Todesfeststellung im Rettungswesen. Notfall Hausarztmedizin 2007;33:330-35.

[61] Ding WY, Meah M, Mann P. Unassisted return of spontaneous circulation after ventricular fibrillation. J Cardiovasc Electrophysiol. 2018;29:199-200.

[62] Lapinsky SE, Leung RS. Auto-PEEP and electromechanical dissociation. N Engl J Med. 1996;335:674.

[63] Dhanani S, Hornby L, Ward R, Baker A, Dodek P, Chamber-Evans J, et al. Vital signs after cardiac arrest following withdrawal of life-sustaining therapy: a multicenter prospective observational study. Crit Care Med. 2014;42:2358-69.

[64] Greer R, Soar J. Lazarus phenomenon: Confirmation of death after unsuccessful cardiopulmonary resuscitation. Resuscitation. 2013;84:e151.

[65] Anonymous. Diagnosis of death after cessation of cardiopulmonary resuscitation. Signal. Reference number 1329, 28 February 2012.: National Archives; 2012. In: [http://webarchive.nationalarchives.gov.uk/20171030124140/http://www.nrls.npsa.nhs.uk/resources/?p=3](http://webarchive.nationalarchives.gov.uk/20171030124140/http:/www.nrls.npsa.nhs.uk/resources/?p=3)

[66] Skulberg A. Criteria of death and time of death. Do Norwegian physicians follow laws and regulations? . Tidsskr Nor Laegeforen 1991;111:3310-11.

[67] Mateos-Rodríguez A, Pardillos-Ferrer L, Navalpotro-Pascual JM. Kidney transplant function using organs from non-heart-beating donors maintained by mechanical chest compressions. Resuscitation 2010;81:904-07.
