# Supplementary material for: Autoresuscitation (Lazarus phenomenon) after termination of cardiopulmonary resuscitation - a scoping review
Source: Scand J Trauma Resusc Emerg Med. 2020 Feb 26;28:14. doi: 10.1186/s13049-019-0685-4 (PMC7045737; doi:10.1186/s13049-019-0685-4)
Supplement: Supplementary file 3 — Additional file 3. Published reviews and prospective studies [file 13049_2019_685_MOESM3_ESM.docx]

## **Supplemental File 1 Published reviews and prospective studies**

Several reviews and prospective studies have been published. It is clear that documentation for individual cases is very variable and sometimes lacking in important information. Nevertheless, certain useful observations can be made.

The first systematic review was performed to address the lack of scientific evidence concerning autoresuscitation and its potential impact on the practice of organ donation after cardiac death.^1^ The authors defined ROSC as heart sounds, pulse (detected by palpation or Doppler ultrasound), blood pressure (detected by invasive or non-invasive methods), oxygenation (detected by pulse oximetry), opening of the aortic valve (detected by echocardiography) and resumption of breathing or neurologic function. Reports documenting the presence of ECG activity without any of the above signs of ROSC were excluded. Twenty-seven papers were identified describing 32 cases from 16 different countries. All of the subjects were adults (age range 27-94y) and all occurred after failed CPR. The rhythms prior to autoresuscitation occurring were asystole (19), PEA (8), VF (2) and Not Recorded/Other (3). There were no cases when CPR was not provided and no cases of autoresuscitation following a planned withdrawal of life-sustaining therapy preceding planned organ donation. The duration of CPR ranged from 6-88min and asystole was the most frequently reported heart rhythm before autoresuscitation (63%). The time interval from failed CPR to unassisted ROSC ranged from “a few seconds” to 33min (mean 7.9min). In eight cases, ECG monitoring had been continued after resuscitation and exact times were recorded. In one case, ROSC occurred at 3min, in six cases at 5min and in one case at 7min. Of the 20 cases for which information on the patient’s level of consciousness was available, 14 (70%) reported a return of consciousness, eight of which recovered fully, with follow-up information ranging from 18 days to 6 months. A ninth patient made a cardiovascular recovery but was neurologically impaired. In the 11 cases that did not demonstrate asystole by ECG, it is impossible to confirm that circulatory arrest had actually occurred and potentially, CPR had been terminated prematurely. Of the 27 cases for which information on the patient’s final outcome was available, 18 (67%) died. Finally, the authors emphasised that the provision of CPR in organ donors after cardiac death has been diagnosed may influence the observation time before organs can be harvested.

The same authors updated their review in 2018, again primarily because of concerns relating autoresuscitation and organ donation after circulatory death.^2^ Since 2010, there were an additional two observational studies, one retrospective,^3^ one prospective,^4^ and one chart review^5^ in patients undergoing withdrawal of life-sustaining therapy prior to organ donation. The combined figures from the two observational studies were six cases of transient autoresuscitation out of a total of 114 patients. In the chart review, transient ECG and arterial pulsation returned in two out of 81 patients. There were also 13 further case reports of autoresuscitation in patients after cessation of CPR rather than withdrawal of life-sustaining therapy (10 adult, three paediatric). The rhythms in the latter cases prior to autoresuscitation were asystole (9); VF (2); PEA (2). The length of CPR time ranged from 15-90min and the duration of circulatory arrest after cessation of CPR until return of ECG and arterial blood pressure tracing or palpable pulse or respiration was reported to be from 30sec to 10min. In cases with continuous monitoring and confirmation of circulation, the longest times before autoresuscitation occurred were 10 and 2min for adults and children respectively. Five of the 13 patients (all adults) had a return of consciousness, and four (31%) survived, three with no or minor impairments and one with minimal neurologic function. Of the three paediatric cases of autoresuscitation, none survived. The authors emphasised that it is important that healthcare professionals are aware of the possibility of autoresuscitation and monitor their patients accordingly before diagnosing death. They concluded that although the articles analysed offer a low level of evidence, they do suggest that it is advisable to monitor CA patients for 5-10min after stopping CPR to confirm asystole before certifying death.

Another systematic review^6^ found 38 articles, published between 1982 and 2012 from 20 different countries. All but four were the same as in the above two reviews. There were 49 patients for which records were available. 51% were male and the mean age was 63.3 years (range 9 months to 94 years). Times from loss of circulation or termination of CPR attempts until spontaneous return ranged from “several seconds” to 33min. Thirty-three patients (67%) recovered circulation within 10min; a further five achieved ROSC between 10-20min and one patient at 33 min, ^7^ although the latter time is misleading as it relates to the time after death that breathing was noticed and not the exact time that autoresuscitation occurred. The commonest cardiac rhythm was asystole (n=30). Three cases were spontaneous auto-defibrillation, without therapy being applied, after CPR had been abandoned in the presence of pulseless ventricular tachyarrhythmia.^8-10^ Thirty-one patients (65%) died while hospitalised and 11 (22%) were discharged alive without complications or with only slight neurologic impairment.

Given the importance of autoresuscitation, a prospective six-year study in Finland (2011-16) of CPR delivered by EMS looked at unsuccessful CPR in which patients were pronounced dead on-scene.^11^ They excluded a few cases of accidental hypothermia who were transported under continuous CPR for invasive rewarming in hospital. CPR was continued for up to 35min if VF and up to 20min with asystole or PEA. All out-of-hospital CPR attempts were carefully monitored for 10min after the cessation of CPR in order to detect delayed ROSC of any duration. This practice was introduced in 2010 following a malpractice case in another part of Finland. Following a 10min monitoring period, patients were declared dead if no signs of life were detected. Of the 2102 out-of-hospital arrests, CPR was attempted in 1376 and was terminated on-scene in 840 cases. Autoresuscitation occurred five times (incidence 5.95/1000) in on-scene-terminated CPR attempts. Time to delayed ROSC from the cessation of CPR varied from 3-8min. The authors pointed out that none of the cases were “classic” out-of-hospital arrests i.e. with strictly guideline-based CPR. Two were elderly nursing-home residents, two were haemorrhagic arrests so fluid resuscitation was a priority over epinephrine administration during CPR and one was carbon monoxide poisoning. Four of the five cases had PEA when CPR was stopped. All patients died (three on-scene; two in hospital).

**References**

[1] Hornby K, Hornby L, Shemie SD. A systematic review of autoresuscitation after cardiac arrest. Crit Care Med. 2010;38:1246-53.

[2] Hornby L, Dhanani S, Shemie SD. Update of a Systematic Review of Autoresuscitation After Cardiac Arrest. Crit Care Med. 2018;46:e268-e72.

[3] Sheth KN, Nutter T, Stein DM, Scalea TM, Bernat JL. Autoresuscitation after asystole in patients being considered for organ donation. Crit Care Med. 2012;40:158-61.

[4] Dhanani S, Hornby L, Ward R, Baker A, Dodek P, Chamber-Evans J, et al. Vital signs after cardiac arrest following withdrawal of life-sustaining therapy: a multicenter prospective observational study. Crit Care Med. 2014;42:2358-69.

[5] Yong SA, D'Souza S, Philpot S, Pilcher DV. The Alfred Hospital experience of resumption of cardiac activity after withdrawal of life-sustaining therapy. Anaesth Intensive Care. 2016;44:605-6.

[6] Peña SB, Aedo IF, Palomino SL. Spontaneous return of circulation after termination of cardiopulmonary resuscitation maneuvers: a systematic review of cases of Lazarus phenomenon. Emergencias 2014;26:307-16.

[7] Puschel K, Lach H, Wirtz S, Moecke HP. Ein weiterer Fall von “Lazarus-Phänomen”? . Notfall Rettungsmedizin. 2005;8:528-32.

[8] Kamarainen A, Virkkunen I, Holopainen L, Erkkila EP, Yli-Hankala A, Tenhunen J. Spontaneous defibrillation after cessation of resuscitation in out-of-hospital cardiac arrest: a case of Lazarus phenomenon. Resuscitation. 2007;75:543-6.

[9] Krarup NH, Kaltoft A, Lenler-Petersen P. Risen from the dead: a case of the Lazarus phenomenon-with considerations on the termination of treatment following cardiac arrest in a prehospital setting. Resuscitation. 2010;81:1598-9.

[10] Gomes E, Araujo R, Abrunhosa R. Two successful cases of spontaneous recovery after cessation of CPR. Resuscitation. 1996;31.

[11] Kuisma M, Salo A, Puolakka J, Nurmi J, Kirves H, Vayrynen T, et al. Delayed return of spontaneous circulation (the Lazarus phenomenon) after cessation of out-of-hospital cardiopulmonary resuscitation. Resuscitation. 2017;118:107-11.
