# Supplementary material for: Autoresuscitation (Lazarus phenomenon) after termination of cardiopulmonary resuscitation - a scoping review
Source: Scand J Trauma Resusc Emerg Med. 2020 Feb 26;28:14. doi: 10.1186/s13049-019-0685-4 (PMC7045737; doi:10.1186/s13049-019-0685-4)
Supplement: Supplementary file 4 — Additional file 4. Published definitions of autoresuscitation [file 13049_2019_685_MOESM4_ESM.docx]

**Appendix 1.** Published definitions of autoresuscitation

- “The Lazarus phenomenon is described as delayed return of spontaneous circulation after cessation of cardiopulmonary resuscitation”^1, 2^
- “The return of spontaneous circulation after failed resuscitation efforts”^3^
- “Delayed return of spontaneous circulation after cardiac arrest when the resuscitation efforts are discontinued”^4^
- “Survival after failed CPR”^5^
- “Spontaneous recovery of circulatory function after unsuccessful CPR”^6^
- “Delayed return of the native circulation [after abandoning resuscitation]”^7^
- “The spontaneous return of circulation subsequent to the termination of resuscitation efforts in a patient suffering from cardiac arrest”^8^

**References**

[1] Maeda H, Fujita MQ, Zhu BL, Yukioka H, Shindo M, Quan L, et al. Death following spontaneous recovery from cardiopulmonary arrest in a hospital mortuary: 'Lazarus phenomenon' in a case of alleged medical negligence. Forensic Sci Int. 2002;127:82-7.

[2] Kuisma M, Salo A, Puolakka J, Nurmi J, Kirves H, Vayrynen T, et al. Delayed return of spontaneous circulation (the Lazarus phenomenon) after cessation of out-of-hospital cardiopulmonary resuscitation. Resuscitation. 2017;118:107-11.

[3] Meeker JW, Kelkar AH, Loc BL, Lynch TJ. A Case Report of Delayed Return of Spontaneous Circulation: Lazarus Phenomenon. Am J Med. 2016;129:e343-e4.

[4] Sukhyanti K, ShriKrishan C, Anu K, Ashish D. Lazarus phenomenon revisited: a case of delayed return of spontaneous circulation after carbon dioxide embolism under laparoscopic cholecystectomy. Anaes Pain Int Care 2012.

[5] Ben-David B, Stonebraker VC, Hershman R, Frost CL, Williams HK. Survival after failed intraoperative resuscitation: a case of "Lazarus syndrome". Anesth Analg. 2001;92:690-2.

[6] Frolich MA. Spontaneous recovery after discontinuation of intraoperative cardiopulmonary resuscitation: case report. Anesthesiology. 1998;89:1252-3.

[7] Bray JG, Jr. The Lazarus phenomenon revisited. Anesthesiology. 1993;78:991.

[8] Spowage-Delaney B, Edmunds CT, Cooper JG. The Lazarus phenomenon: spontaneous cardioversion after termination of resuscitation in a Scottish hospital. BMJ Case Rep. 2017;2017.
